# Investigating the Link Between Intimate Health, Hygiene and Sexual Practices and the Vaginal Microbiome—The INTIMATE Study

**DOI:** 10.1002/rmb2.12685

**Published:** 2025-10-21

**Authors:** Kristina Magoutas, Alexandra Holdcroft, Melanie Walls, Lucy Furfaro, Demelza Ireland, Matthew Payne

**Affiliations:** ^1^ Division of Obstetrics and Gynaecology, Medical School University of Western Australia Perth Western Australia Australia; ^2^ Concept Fertility Perth Western Australia Australia; ^3^ School of Biomedical Sciences University of Western Australia Perth Western Australia Australia

**Keywords:** intimate health, sexual practices, vaginal microbiome

## Abstract

**Purpose:**

To explore how intimate hygiene practices and related products—such as feminine washes, wipes, and lubricants—affect the vaginal microbiome.

**Methods:**

Vaginal bacterial communities of 96 non‐pregnant Western Australian women were characterised via full‐length 16S rRNA gene sequencing. Participants completed an online questionnaire capturing demographics, intimate health, hygiene and sexual practices, to compare these with microbial composition.

**Results:**

Beta diversity varied significantly across multiple variables, particularly in relation to *Lactobacillus* spp. abundance. Compared to women with no partners or two or more sexual partners, monogamous women had lower *L. crispatus* (*p* = 0.002 and *p* = 0.04, respectively), higher *L. iners* (*p* = 0.017 and *p* = 0.044, respectively), and were least likely to cluster into CST I (*p* < 0.001). Postmenopausal women showed reduced *L. crispatus* (*p* = 0.009), increased levels of *L. iners* (*p* = 0.037), and were most likely to cluster into CST IV (*p* = 0.029) compared with women who did not report menopause. Regular perineal washing was associated with fewer reported infections (*p* = 0.029), whereas internal washing correlated with recurrent thrush (*p* = 0.017).

**Conclusions:**

Sexual practices and menopause status are key factors influencing *L. crispatus* and *L. iners* colonisation. Most hygiene practices had minimal impact on Lactobacillus dominance, although behaviours associated with significant microbiome disruption were uncommon in this cohort.

## 1 Introduction

The vaginal microbiome is a dynamic, sensitive microenvironment that changes in response to pregnancy, menstrual cycle, contraceptive use, and diet [1]. The vaginal microbiota lives in a mutualistic relationship with the host, providing protection from pathogenic bacteria in exchange for nutrients and shelter [1, 2, 3]. A significant amount of protection is provided by bacteria from the genus *Lactobacillus* spp., which contributes to the acidic vaginal pH found in most women via the production of lactic acid [1, 3]. However, the levels of protective Lactobacilli can be easily disrupted via a range of external factors, predisposing women to a non‐optimal vaginal state and a variety of adverse vaginal health conditions such as bacterial vaginosis (BV), candidiasis (thrush), and sexually transmitted infections (STIs) [1, 2, 3]. The vaginal microbiome has multiple ‘core microbiomes’, known as Community State Types (CSTs), which all provide differing levels of resistance and resilience to disturbances [4]. CST I, II, III, and V are characterized by dominance of *L. crispatus* , *L. gasseri* , *L. iners* , and *L. jensenii* respectively, while CST IV is diverse relative to the other CSTs and is *Lactobacillus* spp. depleted [4]. Although in most cases, high levels of *Lactobacillus* spp. (CST I, II, III, V) and a pH < 4.5 are synonymous with ‘health’, many women who are CST IV can be asymptomatic, especially Black and Hispanic women who are overrepresented in this CST [4, 5, 6] Hence, a holistic approach to characterization of the vaginal microbiome that merges CST profiles with relevant clinical, social, and environmental variables and, in particular, inflammatory states, needs to be adopted when attempting to interpret health and disease state [7].

The term ‘intimate hygiene practices’ encompasses a wide variety of practices and associated products used to cleanse and groom in and/or around the genital area. The most well‐studied vaginal hygiene practice, douching, involves the introduction of water and/or cleansing products into the vagina. Douching has been associated with increased risks for BV, preterm birth, and pelvic inflammatory disease (PID), the latter of which can lead to infertility [8, 9]. It has been hypothesized that douching alters the microbial community within the vagina, causing inflammation and providing an opportunity for pathogenic bacteria to flourish [10]. Aside from vaginal douching, little is known of the effect that other intimate hygiene products such as gels, sprays, and wipes have on the vaginal microbiome. Furthermore, previous studies that have attempted to document the impacts of intimate hygiene product use on vaginal health in general have some core limitations.

The term ‘vagina’ is used in some studies as an all‐encompassing term for the genital area. For example, Sabo et al. used the term ‘vaginal washing’ but failed to explain which specific products or formulations were used and whether participants considered the vagina to be the internal organ, or a term used to describe the entire female genitalia [11]. This distinction is important as the vulva and vagina are two distinctly different areas with unique microbial environments [10]. Without thorough documentation of the internal and external use of hygiene products via detailed questionnaires, we cannot be certain of the true usage rates of specific products and practices and therefore their impact on the vaginal microbiome.

The most robust assessment of intimate hygiene practices to date, conducted by Crann et al. [12], documented hygiene product usage and practices in Canadian women using a detailed questionnaire. Associations between hygiene product use and adverse vaginal conditions were found, but links between this and the vaginal microbiome were not investigated as they did not conduct paired bacterial profiling analyses [12]. In contrast, whilst Sabo and Balkus [11] conducted a thorough bacterial profiling analysis of the vaginal microbiome, they failed to couple these data with a questionnaire or adequate description of feminine hygiene product usage and practices within the cohort.

In the current study, we collected mid‐vaginal swabs, matched with detailed questionnaire data on feminine hygiene practices, sexual behavior, and contraceptive use. Using this, we aimed to document vaginal bacterial profiles among non‐pregnant women in Western Australia and describe any associations between participant behaviors and practices, the vaginal microbiome, and adverse vaginal health conditions.

## 2 Materials and Methods

### 2.1 Study Design

This prospective cohort study consisted of 96 non‐pregnant women recruited between June and August 2022, either in person from the University of Western Australia or via word of mouth. Women were eligible to participate based on the following criteria: aged > 18 years, not pregnant, and not currently menstruating at the time of sampling. Women who had recently used antibiotics (< 2 weeks) or had engaged in sexual intercourse in the 24 h prior to sampling were asked to return at a later eligible date. The study was approved by the University of Western Australia Human Research Ethics Committee (2022/ET000242). All participants were required to provide written, informed consent. Consenting participants completed an intimate health and hygiene questionnaire and self‐collection of a mid‐vaginal swab for microbial DNA analysis. The sequencing data generated and analyzed in this study are publicly available in the NCBI Sequence Read Archive (SRA) under the accession number [PRJNA1293858]. The raw FASTQ files can be accessed directly through the SRA platform. No restrictions apply to the data, and it is openly available for reuse.

### 2.2 Questionnaire

An intimate hygiene questionnaire was developed based on a similar survey designed by Crann and Cunningham [12], with further local community consultation. Feedback was received from two women. The revised questionnaire (Appendix S1) was administered by Qualtrics in an online format and consisted of 46 close‐ended questions across three sections: intimate hygiene practices, sexual activity, and health practices and diagnoses known to influence the vaginal microbiome. Behavioral questions were presented on a Likert scale from NEVER to DAILY. Sexual activity questions referred to the frequency of use of sexual products and practices, while health questions addressed the frequency and types of adverse vaginal symptoms, as well as diagnoses of urogenital infections such as urinary tract infections (UTIs), Candidiasis (thrush), BV, and STIs in the last 12 months. To estimate the menstrual cycle phase at the time of sampling, we used participants' responses to two questionnaire items: (1) the number of days since their last period, and (2) typical period duration. The period duration responses were categorical: 2 days, 3–5 days, 5–7 days, 7+ days. For each participant, the minimum and maximum cycle days at sampling were calculated by adding the number of days since their last period to the minimum and maximum period duration, respectively.

Cycle day ranges were then used to assign participants to one of three phases:

– Follicular: day 6–12
– Ovulatory: day 12–16
– Luteal: day 17–42

Participants whose cycle day ranges spanned multiple phases or fell outside the standard 28‐day cycle were initially classified as “Uncertain”. Manual overrides were then applied in a small number (*n* = 7) of these cases where cycle ranges fell into transitional periods; in such cases participants were placed into the phase that was about to begin.

### 2.3 Sample Collection and Processing

Women were provided with written, pictorial, and verbal instructions on self‐collection of a mid‐vaginal swab. They were provided with an E‐swab collection kit (Copan Diagnostics, Brescia, Italy) and asked to self‐collect samples in the privacy of the nearest bathroom. Briefly, participants inserted the swab 5 cm into the vagina and rotated this for 20 s, ensuring contact with the walls of the vagina. Swabs were placed immediately into a collection tube containing approximately 1 mL of liquid Amies media, snapped at the mid‐stem breakpoint, and then capped. Swabs were then stored on ice prior to transport to the Clinical Perinatal Research Laboratories at King Edward Memorial Hospital (KEMH) where they were processed within 24 h. Swabs were vortexed for 10 s and the swab head pressed against the tube wall to release any remaining free liquid before being discarded. Vaginal swab eluates were then transferred to DNA‐free 2 mL microfuge tubes (Sarstedt Inc., Nümbredt, Germany) and stored at −80°C for downstream DNA extraction.

### 2.4 DNA Extraction and Quantification

DNA extraction was conducted as per Cheema et al. [13]. Briefly, vaginal swab eluates were defrosted at room temperature and centrifuged at 40 000 × g for 5 min at 4°C. Supernatants were subsequently removed until ~50 μL remained to ensure the cell pellet was left undisturbed. Pellets were resuspended in 353 μL of buffer MBL/ribonuclease A (QIAGEN, Hilden, Germany), transferred to 2 mL microfuge tubes containing 0.1 mm glass beads, and subjected to bead beating for 45 s at 6500 RPM on a Precellys 24 tissue homogenizer (Thermo Fisher Scientific). DNA was extracted using the QIAGEN MagAttract Microbial DNA kit (QIAGEN) on an automated Kingfisher Flex platform (Thermo Fisher Scientific, Waltham, MA), as per the manufacturer's instructions. Two negative extraction controls, each consisting of E‐swabs briefly exposed to the air and processed as per the clinical samples, were included in each run. Extracted DNA was stored at 4°C until PCR and sequencing analysis.

### 2.5 16S rRNA Gene Amplification and Barcoding

DNA from 95 vaginal swabs was analyzed, with one omitted due to an insufficient sample after DNA extraction. The full‐length 16S rRNA gene was PCR amplified using the primer pairs 27F (5′AGRGTTYGATYMTGGCTCAG‐3′) and 1492R (5′‐RGYTACCTTGTTACGACTT‐3′). PCRs were conducted in 25 μL reactions containing 0.3 μM each of unique, asymmetrically barcoded forward and reverse primers, 1 X Accustart II Toughmix (Quantabio, Beverley, MA, USA), 0.75 μL each of ArcticZymes dsDNase and DTT (ArcticZymes PCR de‐contamination kit, Trømso, Norway), 5.5 μL nuclease‐free water, and 5 μL of template or nuclease‐free water (PCR negative controls). Our 16S rRNA gene amplification method was adapted from Cheema et al. [13]; however, we did not perform a second barcoding step, instead using forward and reverse barcoded primers in a single PCR reaction. Two negative template controls were included for every 94 samples. PCR cycling conditions were: 94°C for 3 min, 32 cycles of 94°C for 30 s, 52°C for 30 s, 72°C for 2 min, and a final extension step of 72°C for 5 min. Any samples that produced little to no amplicon were re‐run as above using 40 cycles. PCR products were visualized on a QIAxcel capillary gel electrophoresis system (QIAGEN) using a DNA high‐resolution cartridge to confirm the presence, size, and quantity of amplicons. Barcoded PCR amplicons were pooled in equimolar concentrations based on the QIAxcel quantitation of the target band (~1500 bp). The amplicon pool was made up to 400 μL in nuclease‐free water (Integrated DNA Technologies) and purified using Nucleomag NGS magnetic beads (Macherey‐Nagel, Düren, Germany), using a 0.6× ratio as per the manufacturer's instructions.

### 2.6 PacBio HiFi Sequencing

Purified amplicon pools were sent to the Australian Genome Research Facility (AGRF) at the University of Queensland for library preparation and sequencing. SMRTbell adapters were ligated onto the barcoded PCR products, and libraries were sequenced by high‐fidelity (HiFi) sequencing on a single SMRT cell using the PacBio Sequel II system. Raw data were processed using PacBio SMRTlink to generate demultiplexed fastq files.

### 2.7 Data Analysis

Demultiplexed sequence data were processed using Mothur version 1.48.0. Sequences outside the biological length of the 16S rRNA gene (1336–1743 bp) and those containing homopolymers > 9 bases were removed. Alignment was performed using the SILVA reference alignment database (v138). Chimeric sequences were identified using VSEARCH and removed. OTU clustering was performed with a similarity cut‐off of 0.03. Taxonomic identification of OTUs was generated using BLAST with a cut‐off of > 99% sequence identity and 99% sequence coverage at the species level. Taxonomic assignments for OTUs identified in negative extraction and PCR controls are reported in Table S1. Vaginal microbial profiles were clustered into one of five community state types (CSTs) as per the original scheme proposed by Ravel, Gajer [4].

All microbiome analyses were performed on subsampled data. Unless otherwise stated, all compositional analyses were performed at the OTU level. OTUs which made up an average relative abundance of > 1% were included in analyses. Differential abundance analyses were performed on untransformed data. Alpha diversity was measured using OTU richness and Shannon diversity. Beta diversity was measured using Bray–Curtis distance.

The effect of specific vaginal hygiene practices on microbiome community structure was assessed by PERMANOVA using the adonis2 function of the R package vegan with 9999 permutations. For statistically significant variables containing greater than two data points (e.g., non‐binary variables), pairwise PERMANOVA was utilized to assess the contribution of each variable. All significant data were visualized using non‐metric multi‐dimensional scaling (NMDS) star plots using the R package ggplot2.

Differential abundance analyses were conducted on all significant variables using the Metastats function within Mothur. Metastats outputs were filtered to show which OTUs were significantly different within groups being compared, and these were subsequently visualized using jitter/box and whisker plots using the R package ggplot2.

Associations with vaginal hygiene practices were analyzed using Chi‐square tests, or when expected cell frequencies were low for categorical outcomes, Fisher's exact tests.

Descriptive summaries of *Lactobacillus* spp. counts, vaginal community state types (CST), and adverse vaginal health conditions such as STIs, UTIs, and thrush were made using frequency distributions. SPSS statistical software and R studio were used in data analysis.

## 3 Results

### 3.1 Participant Health and Hygiene Characteristics

The majority of study participants were Caucasian (93.8%) and aged between 18 and 29 years (54.2%) (Table 1). Most participants (82.3%) reported following no particular diet.

**TABLE 1:** Participant demographic characteristics.

Characteristic	*N* (%)
Age, year	
18–29	52 (54.2)
30–39	15 (15.6)
40–49	14 (14.6)
50+	15 (15.6)
Race or ethnicity	
Caucasian	90 (93.8)
Asian	3 (3.1)
Aboriginal	1 (1.0)
Mixed race	2 (2.1)
Diet	
Vegetarian	11 (11.5)
Vegan	4 (4.2)
Paleo	1 (1.0)
Low FODMAP	1 (1.0)
None	79 (82.3)

Abbreviation: FODMAP, Fermentable Oligosaccharides, Disaccharides, Monosaccharides, and Polyols.

#### 3.1.1 Intimate Hygiene Product Use and Practices

Participants reported using a range of hygiene products and practices (Table 2). External cleansing was reported by over 90% of participants. Only 18% of participants reported internal washing. Water only was the most common cleansing product (88% and 86% of those who cleansed internally and externally, respectively). Many women reported the use of soaps and shower gels, with the majority using these products externally. Intimate washes, commercially manufactured products designed and marketed specifically for the intimate area, were used by 23.5% and 10.3% of those washing internally and externally, respectively. No participants reported vaginal douching or vaginal steaming. Facial and baby wipes, not specifically designed for the intimate area, were used by 11.5% of those cleansing externally, with most women using these products monthly. Participants also reported ‘other’ products used but not specifically listed on the survey; these included pH‐neutral or soap‐free products. Almost 90% of participants reported pubic grooming, with shaving (68.6%) and trimming (45.3%) the most common methods.

**TABLE 2:** Participant intimate hygiene practices (*n* = 96).

Internal (vaginal) cleansing	*N* (%)
Yes	17 (17.7)
No	78 (82.3)

		Daily	Multiple times a week	Weekly	Monthly	Never
Internal cleansing products (if yes to internal cleansing)						
Water only	15 (88.3)	14 (82.4)	1 (5.9)	0	0	2
Soap ± water	6 (35.3)	3 (17.7)	1 (5.9)	1 (5.9)	1 (5.9)	11
Shower gel	3 (17.6)	1 (5.9)	1 (5.9)	0 (0)	1 (5.9)	14
Intimate washes	4 (23.5)	2 (11.8)	0	1 (5.9)	1 (5.9)	13
Intimate wipes	0	0	0	0	0	17
Intimate powders	0	0	0	0	0	17
Cleansing wipes	0	0	0	0	0	17
Moisturizers	0	0	0	0	0	17
Talcum powder	1 (5.9)	0	0	0	1	16
Vaginal douching	0	0	0	0	0	17
Vaginal steaming	0	0	0	0	0	17
Other	1 (5.9)	1 (5.9)	0	0	0	16
External (vulval) cleansing						
Yes	87 (90.6)					
No	9 (9.4)					
External cleansing products (if yes to external cleansing)						
Water only	75 (86.2)	62 (70.5)	11 (12.5)	2 (2.3)	0	12
Soap ± water	49 (56.3)	25 (28.8)	14 (16.1)	3 (3.5)	7 (8.1)	38
Shower gel^a^	30 (34.5)	18 (20.5)	5 (6.8)	3 (3.4)	4 (4.6)	57
Intimate washes	9 (10.3)	3 (3.5)	4 (4.6)	0	2 (2.3)	78
Intimate wipes	3 (3.4)	0	1 (1.2)	0	2 (2.3)	84
Intimate powders	1 (1.2)	0	0	0	1 (1.2)	86
Cleansing wipes	10 (11.5)	0	1 (1.1)	2 (2.3)	7 (8.1)	77
Moisturizers	6 (6.9)	0	1 (1.2)	1 (1.2)	4 (4.6)	81
Intimate deodorant	1 (1.2)	0	0	0	1 (1.2)	86
Other	8 (9.2)	6 (6.9)	1 (1.1)	0	1 (1.2)	79
Regular perineum cleansing						
Yes	68 (71.6)					
No	27 (28.4)					
Pubic grooming						
Yes	86 (90.5)					
No	9 (9.4)					
What methods in the previous 6 months?^b^ (If yes to pubic grooming)						
Shaving with soap/shower gel		59 (68.6)				
Shaving with cream/gel		12 (13.9)				
Waxing		22 (25.6)				
Laser/IPL		12 (13.9)				
Hair removal cream		11 (12.8)				
Trimming		39 (45.3)				
Bleaching/dying		0				
Underwear to bed						
Yes	64 (66.7)					
No	32 (33.3)					
Synthetic underwear						
Yes	18 (18.8)					
No	78 (81.2)					

^a^ 1 response removed due to selecting both multiple times per week and never.

^b^ Participants were instructed to select all that apply.

#### 3.1.2 Sexual Practices

Sexual practices that might influence the vaginal microbiome are described in Table 3. Most women were in monogamous relationships (63.5%), had been sexually active in the past 12 months (79.2%), and had one sexual partner during this time (76.3%). The most common sexual practice was vaginal intercourse, followed by fingering and oral sex. Lubricant was used by 48% of women and was used most of the time during sexual activity.

**TABLE 3:** Participant sexual practices.

Characteristic	*N* (%)
Monogamous relationship	
Yes	61 (63.5)
No	35 (36.5)
Sex in the past 12 months	
Yes	76 (79.2)
No	20 (21)

Number of sexual partners	0	1	2+
	20 (21)	57 (60)	18 (19)

Sexual practices		Daily	Multiple times a week	Weekly	Monthly
Vaginal intercourse	79 (82.2)	0	25 (30.9)	25 (30.9)	29 (35.8)
Anal intercourse	7 (9.2)	0	0	1 (1.4)	6 (8.1)
Oral sex (receiving)	61 (80.3)	0	8 (10.7)	14 (18.7)	39 (52.0)
Fingering	68 (89.5)	0	16 (20.8)	22 (28.6)	30 (40.0)
Use of sex toys	41 (54.0)	0	7 (9.3)	11 (14.7)	23 (30.7)

Lubricant use		Every time	Most times	Sometimes	Rarely
	46 (47.9)	8 (17.4)	19 (41.3)	14 (30.4)	5 (10.9)

#### 3.1.3 Contraceptive Use and Menopause

Over 50% of women reported using contraception (Table 4). Male condoms were the most common barrier method used (57% of contraceptive users). Only one respondent reported the use (rarely) of a female condom. No participants used diaphragms or spermicides. Hormonal contraceptive use was reported by 41 women, with an intrauterine device (IUD) or combined oral contraceptive pill (COCP) used by 46.3% and 36.6% of these women, respectively. 14 women had reached menopause; five of these were using menopause hormone therapy. 13.5% of participants reported having perimenopausal symptoms such as hot flushes and irregular periods.

**TABLE 4:** Participant contraception and menopause status.

Characteristic	*N* (%)
Contraception use	
Yes	51 (53.1)
No	45 (46.9)

		Every time	Most times	Sometimes	Rarely
Male condom	29 (56.9)	9 (18.0)	4 (8.0)	9 (18.0)	7 (14.0)
Hormonal contraception	41 (42.7)				
Combined oral contraceptive pill (COCP)	15 (36.6)				
Intrauterine device (IUD)	19 (46.3)				
Implant (Implanon)	6 (14.6)				
Progestogen only contraceptive pill	1 (2.4)				

Menopause status	
Yes	14 (13.6)
No	82 (86.4)
Perimenopausal symptoms	11 (13.4)
Menopause hormone therapy	5 (20.0)

#### 3.1.4 Menstruation and Menstrual Hygiene Management

The menstrual characteristics of participants are reported in Table 5. Over 50% of participants reported having a regular period, with 28% reporting that they skipped their periods using hormonal contraception. Most women were in the luteal phase (53.3%). Women self‐reported their average period flow, with moderate and heavy flow experienced by 44.4% and 27.1%, respectively. Most participants reported periods lasting between 3 and 7 days. Tampons were the most common period product used (82.9%), followed by sanitary pads and period underwear.

**TABLE 5:** Participant menstrual cycles and management.

Characteristic	*N* (%)					
**Regular periods**		**Yes**	**No**	**No, skip using hormonal contraception**		
	82	45 (54.9)	14 (17.1)	23 (28.1)		
**Typical period flow**		**Light**	**Moderate**	**Heavy**		
	81 (84.4)	23 (28.4)	36 (44.4)	22 (27.1)		
**Average period duration**		**2 days**	**3–5 days**	**5–7 days**	**7+ days**	
	81 (84.4)	3 (3.7)	44 (54.3)	27 (33.3)	7 (8.4)	
**Menstrual phase during sampling** (**days since last period**)		**Follicular (6–12)**	**Ovulation (12–16)**	**Luteal (17–42)**		
	45 (46.8)	12 (26.7)	9 (20)	24 (53.3)		

Period product use		Every time	Most times	Sometimes	Rarely	Never
Sanitary Pads	63 (76.9)	27 (35.1)	13 (16.9)	16 (20.8)	7 (9.1)	14
Tampons	68 (82.9)	29 (35.4)	16 (19.5)	11 (13.4)	12 (14.6)	14
Period Cups	16 (19.5)	5 (6.5)	3 (3.9)	5 (6.5)	3 (3.9)	61
Period Underwear	36 (43.9)	15 (19.2)	10 (12.8)	5 (6.4)	6 (7.7)	42

#### 3.1.5 Adverse Vaginal Conditions and Symptoms

A total of 22% of women reported regularly experiencing adverse vaginal symptoms (Table 6). Due to low‐frequency counts for individual symptoms, these are reported as a single item. Self‐reported vaginal health conditions most experienced in the past twelve months included UTIs and Candidiasis by 30.2% and 27.1% of participants, respectively. Notably, 17 participants reported two or more UTIs in the past year.

**TABLE 6:** Adverse vaginal symptoms and health conditions.

Characteristics	*N* (%)
Adverse vaginal symptoms (itching, burning, abnormal discharge)	
Yes	21 (21.1)
No	75 (78.9)

Vaginal health conditions (last 12 months)		Once	2+ times	No
Sexually transmitted infection (STI)	3 (3.1)	0	3 (3.13)	93
Bacterial vaginosis (BV)	9 (9.4)	6 (6.3)	3 (3.13)	87
Urinary tract infection (UTI)	29 (30.2)	12 (12.5)	17 (17.7)	67
Candidiasis (thrush)	26 (27.1)	17 (17.7)	9 (9.4)	70

Iron deficiency diagnosis	
Yes	59 (61.5)
No	37 (38.5)

#### 3.1.6 Participant Antibiotics, Probiotics and Supplement Use

40.6% of participants had used antibiotics in the past year; however, we did not enquire as to the clinical indication for use. Over 60% of participants reported having ever been diagnosed with iron deficiency, and 17% were currently using iron supplements. 15% of participants reported regular probiotic use (Table 7).

**TABLE 7:** Participant antibiotics, probiotics, and supplement use.

Product (*N* = 96)	Yes	Once	2+ times	No
Antibiotic use	39 (40.6)	20 (20.8)	19 (19.8)	57
OTC thrush treatment	—	12 (12.5)	11 (11.4)	73
Iron supplements	16 (16.7)	—	—	80
Probiotics (oral or vaginal)	13 (13.5)	—	—	83

### 3.2 Associations Between Participant Intimate Health, Hygiene and Sexual Practices and Adverse Vaginal Symptoms and Conditions

Due to low frequencies for individual conditions, vaginal conditions (STI, thrush, BV, and UTI) were collapsed into one variable and analyzed based on the presence or absence within the previous year. Regular washing of the perineum was a more common practice among women who did not report any vaginal conditions (80.8% vs. 60.5%, *p* = 0.029) (Table 8). Although the numbers were small, women who reported recurrent thrush (defined as two or more episodes of thrush in the past year) more commonly washed internally (57.1% vs. 14.6%, *p* = 0.017) (Table 8). It is also important to note that although the number of women who washed internally was minimal (*n* = 17), they were twice as likely to report a vaginal condition with this trending towards significance (25.6% vs. 11.5%, *p* = 0.076). There were no statistically significant associations between external washing, contraceptive use, or sexual practices and vaginal conditions.

**TABLE 8:** Associations between feminine health, hygiene and sexual practices and vaginal conditions.

Characteristic	Vaginal condition *N* = 44	No vaginal condition *N* = 51	*p*	Recurrent thrush *N* = 9^a^	No recurrent thrush *N* = 86	*p*	Vaginal symptoms *N* = 20	No vaginal symptoms *N* = 75	*p*
Health and hygiene practices									
Internal washing	11 (25.6)	6 (11.5)	0.076	4 (57.1)	13 (14.6)	**0.017**	7 (33.3)	10 (13.3)	0.050
External washing	41 (95.3)	45 (86.5)	0.177	8 (88.8)	78 (90.1)	0.510	18 (85.7)	69 (92.0)	0.405
Perineum cleansing	26 (60.5)	42 (80.8)	**0.029**	6 (66.7)	62 (72.1)	0.710	15 (71.4)	54 (72.0)	0.959
Pubic grooming	38 (88.4)	47 (90.4)	0.751	8 (88.8)	78 (90.1)	0.549	19 (90.5)	67 (89.3)	0.880
Underwear to bed	29 (67.4)	34 (65.4)	0.833	5 (55.6)	58 (67.4)	0.479	14 (70.0)	49 (65.3)	0.794
Synthetic underwear	10 (23.3)	8 (15.4)	0.330	2 (22.2)	15 (17.4)	0.066	3 (15.0)	14 (18.6)	1.000
Hormonal contraception	26 (60.5)	24 (46.2)	0.164	4 (44.4)	36 (41.9)	1.000	12 (60.0)	28 (37.3)	0.079
Sexual practices									
Sexually active									
Yes	37 (84.1)	38 (74.5)	0.374	7 (77.8)	68 (79.1)	1.000	18 (90.0)	57 (76.0)	0.225
No	7 (15.9)	13 (25.5)		2 (22.2)	18 (20.9)		2 (10.0)	18 (24.0)	
Sexual partners									
None	7 (15.9)	13 (25.5)	0.125	2 (22.2)	18 (20.9)	0.894	2 (10)	18 (24.0)	0.096
One	25 (56.8)	32 (62.7)		5 (55.6)	52 (60.4)		11 (56.7)	46 (61.3)	
Two or more	12 (27.3)	6 (11.8)		2 (22.2)	16 (18.7)		7 (33.3)	11 (14.7)	
Vaginal intercourse									
Daily	0	0	0.213	0	0	1.00	0	0	0.929
Multiple times per week (2)	15 (34.1)	8 (15.7)		2 (22.2)	21 (24.4)		6 (30.0)	17 (22.7)	
Weekly (3)	9 (20.5)	14 (27.5)		2 (22.2)	21 (24.4)		5 (20.0)	18 (24.0)	
Monthly (4)	13 (29.5)	15 (29.4)		3 (33.3)	25 (29.1)		7 (35.0)	21 (28.0)	
Lubricant user	20 (46.5)	25 (48.1)	0.879	3 (33.3)	42 (48.8)	0.491	12 (60)	33 (44.0)	0.307
Condom user	13 (29.5)	16 (31.4)	0.935	2 (22.2)	27 (31.4)	0.637	6 (30.0)	23 (30.7)	0.301

^a^ Recurrent thrush = 2 or more episodes of Candidiasis within the past year. Bold values indicate statistically significant results at *P* < 0.05.

There were no statistically significant associations between feminine hygiene and sexual practices and the presence of adverse vaginal symptoms among participants. However, women who washed internally were 2.5 times more likely to report adverse vaginal symptoms, bordering on significance (*p* = 0.05) (Table 8). Additionally, women who reported having multiple sexual partners in the past 6 months were 2.25 times more likely to report adverse vaginal symptoms, also trending towards significance (*p* = 0.064).

### 3.3 Vaginal Microbiome Profiling

The total number of high‐fidelity (HiFi) reads was 3 342 024, the median read quality score was 34, and the average number of HiFi passes was 18. The average non‐chimeric read count per sample was 19 794, with reads ranging between 3430 and 44 728. Subsampling was performed to 4780 reads, resulting in an average Good's coverage value of 92.41%. This resulted in the exclusion of a single low‐yield sample, leaving 95 for analysis. Extraction and PCR controls were either completely negative or contained extremely low numbers of bacterial reads; extraction controls 2, 3, and 4 had only 2, 3, and 97 full‐length reads, respectively (Table S1).

**FIGURE 1:**
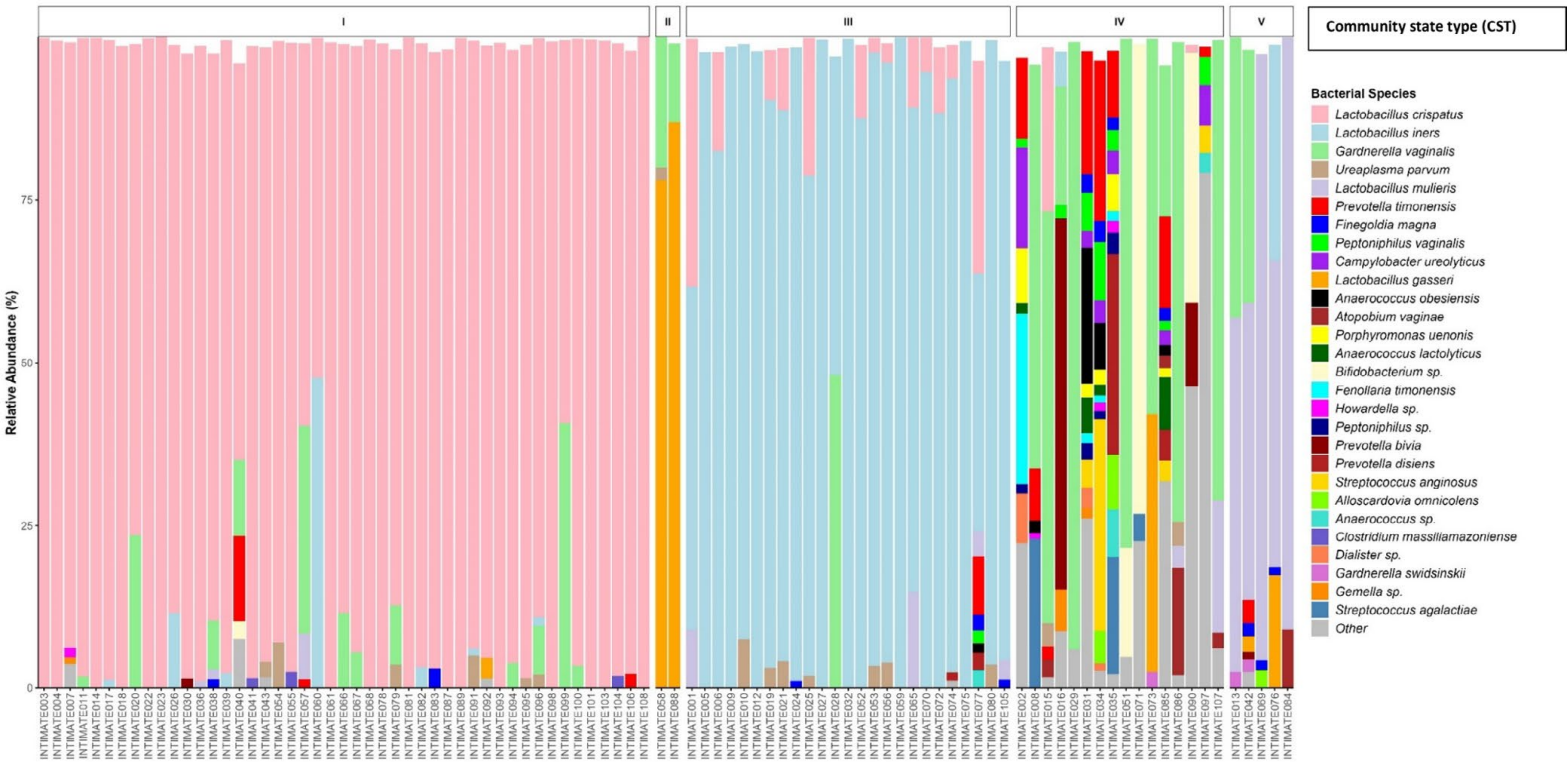
Relative abundance of the 27 most dominant microbial taxa present in 95 participants (INTIMATE 001–108 across the bottom of the figure). Each color represents a microbial taxon, those bacteria which appeared in less than 3 participants were classed as “Other” (color key is indicated on the right‐hand side). Facets represent the community state types (CST I – CST V). The legend is in descending order of occurrence across samples *Lactobacillus crispatus* being the most frequent and *Streptococcus agalactiae* being the least frequent.

In addition to the dominant *Lactobacillus* spp. associated with CST I, II, III, and V, we identified many reads associated with *L. paragasseri* and *L. mulieris*; newly described *Lactobacillus* spp. that are found to be genetically related to *L. gasseri and L. jensenii*, respectively. As a result, we included these species in CST II and V, respectively. In our cohort, 49.5% of participants had a vaginal microbiome consistent with CST I, 26.3% clustered into CST III, and 15.8% were assigned to CST IV. OTUs which occurred in at least 3 participants are represented in Figure 1 in descending order of occurrence. The most frequently occurring bacteria were *L. crispatus* , *L. iners* , *G. vaginalis* , *U* . *parvum*, and *L. mulieris*.

#### 3.3.1 Associations Between Participant Demographic, Intimate Health, Hygiene and Sexual Practices and Community State Types (CSTs)

Table 9 summarizes CST distribution and associated variables. Due to low numbers of participants in CST II and V, they were consolidated into ‘other’ for these analyses.

**TABLE 9:** Associations between participant variables and community state types (CSTs).

Characteristic	CST I	CST II	CST III	CST IV	CST V	Other^a^	*p*
Number of sexual partners in the last 6 months—*N* (%)							
None (*N* = 20)	15 (75)	0	2 (10)	3 (10)	0	0	**0.002**
One (*N* = 57)	18 (31.6)	2 (3.5)	20 (35.1)	12 (21.1)	5 (8.7)	7 (12.2)	
Two or more (*N* = 18)	14 (77.8)	0	3 (16.7)	1 (2.8)	0	0	
Menopause							
True (*N* = 13)^b^	2 (15.4)	1 (7.6)	4 (30.8)	6 (46.2)	0	1 (15.4)	**0.007**
False (*N* = 82)	45 (54.9)	1 (1.2)	21 (25.6)	10 (12.2)	5 (6.1)	6 (7.1)	

^a^ Other = CST II (*n* = 2), CST V (*n* = 5).

^b^ One participant who reported menopause in the questionnaire was removed due to an insufficient amount of sample post‐DNA extraction. Bold values indicate statistically significant results at *P* < 0.05.

CST distribution was associated among women with different numbers of sexual partners in the last 6 months (*p* = 0.002). Post hoc comparison indicated that CST I was significantly more prevalent among women reporting no sexual partners (OR = 3.97, 95% CI: 1.21–15.47, adjusted *p* = 0.049) and two or more partners (OR = 4.59, 95% CI: 1.29–20.94, adjusted *p* = 0.049), and significantly less prevalent in monogamous women (OR = 0.15, 95% CI: 0.05–0.40, adjusted *p* < 0.001). CST III showed a non‐significant trend towards monogamous women (OR = 3.52, 95% CI: 1.12–13.39, adjusted *p* = 0.057). No significant differences were observed for CST IV or the “other” CST category.

CST distribution was also significantly different among women who had reached menopause compared to those who had not (*p* = 0.007) (Table 9). Post hoc comparison showed that CST IV was significantly more prevalent among menopausal women (OR = 6.0, 95% CI: 1.38–26.14, adjusted *p* = 0.029), whereas CST I was significantly less prevalent in menopausal women (OR = 0.15, 95% CI: 0.02–0.76, adjusted *p* = 0.029). No significant differences were observed between CST III or ‘other’ CST categories.

There were no significant associations between hygiene practices and CSTs among participants.

#### 3.3.2 Associations Between Participant Demographic, Intimate Health, Hygiene and Sexual Practices and the Vaginal Microbiome

**FIGURE 2:**
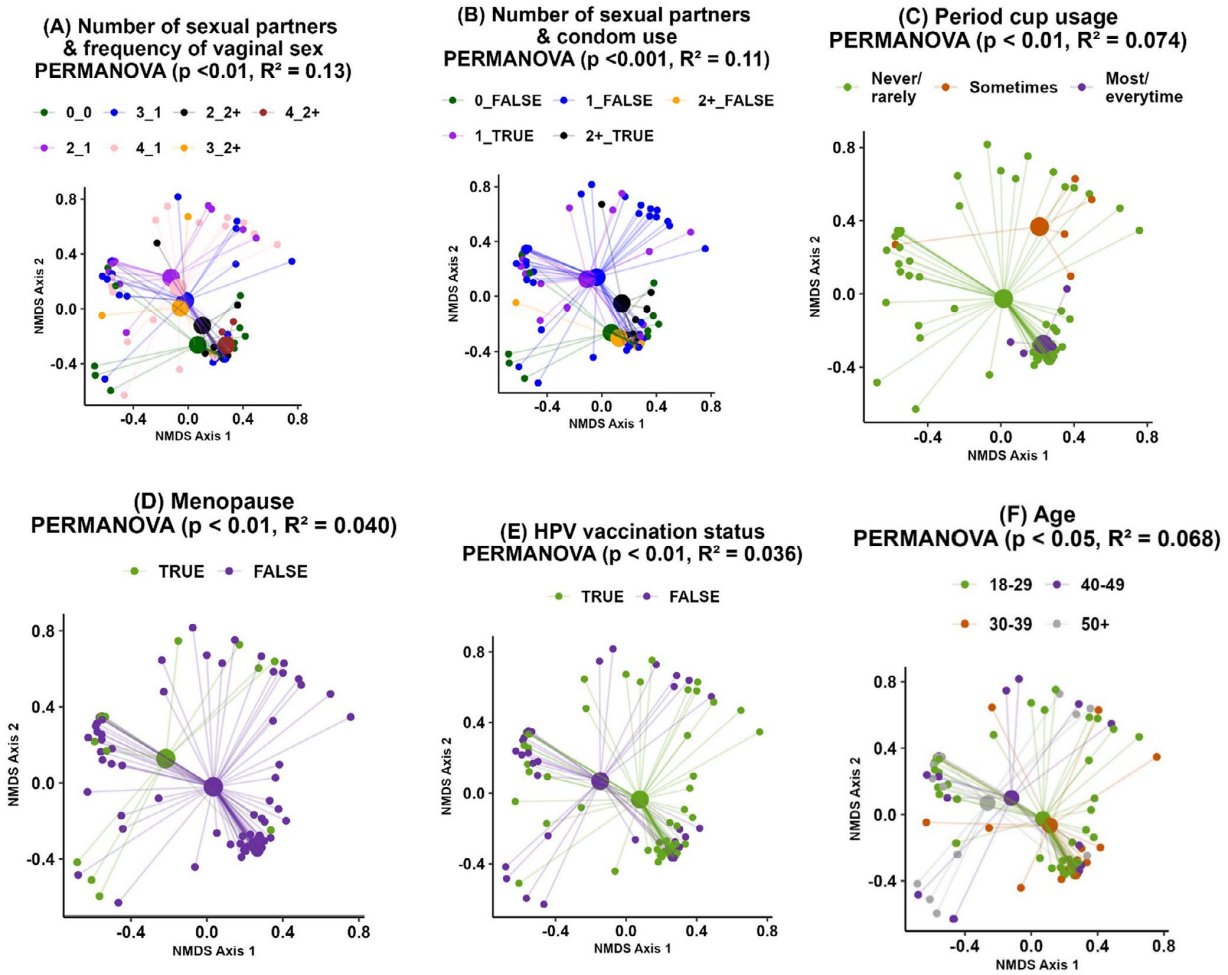
Differences in beta diversity (Bray Curtis index) between samples for variables where significant associations were identified using non‐metric multidimensional scaling (NMDS). The centroid represents the mean distance between samples within the group, while the star points represent individual samples. (A) Number of sexual partners and frequency of vaginal sex interaction (*p* < 0.01); frequency of vaginal sex (0 = no vaginal sex, 2 = multiple times per week, 3 = weekly, 4 = monthly)–number of sexual partners, (B) number of sexual partners and condom use interaction (*p* < 0.001), (C) use of period cups (*n* = 51, *p* < 0.05), (D) menopause status (*p* < 0.001), (E) human papilloma virus (HPV) vaccination status (*p* < 0.01), and (F) age (*p* < 0.05).

Significant differences in vaginal microbiome beta diversity among women were associated with the following variables (Figure 2A–F):

- Number of sexual partners and frequency of vaginal sex interaction (*p* < 0.01, *R* ^2^ = 0.13); frequency of vaginal sex (0 = no vaginal sex, 2 = multiple times per week, 3 = weekly, 4 = monthly) _ number of sexual partners.
- Number of sexual partners and condom use interaction (*p* < 0.001, *R* ^2^ = 0.11)
- Period cup usage in menstruating women; rarely/never, sometimes, and most/every time (*p* < 0.01, *R* ^2^ = 0.074)
- Menopause (*p* < 0.001, *R* ^2^ = 0.04)
- Human papilloma virus (HPV) vaccination (*p* < 0.01, *R* ^2^ = 0.036)
- Age (*p* < 0.05, *R* ^2^ = 0.068)

Due to low numbers of participants in the original categories for sexual partners (1, 2, 3, 4, and 5+), they were consolidated into three groups: zero, one, and two or more partners. Similarly, use of period cups was also consolidated into three groups, as seen above.

Individually, the number of sexual partners (*p* < 0.001, *R* ^2^ = 0.09) and frequency of vaginal sex (*p* < 0.05, *R* ^2^ = 0.026) had significant effects on the vaginal microbiome. However, as we believed it was highly likely that women in monogamous relationships would be engaging in more frequent vaginal intercourse, we then explored the interaction between this and the number of sexual partners (Figure 2A). Despite our assumption, for the most part, clear clustering was evident based on the number of partners but not frequency of vaginal sex.

To further explore potential explanations for the differences seen in beta diversity related to numbers of sexual partners, we ran chi‐square tests to determine if there were any associated participant demographic characteristics or sexual practices. Of note, age and the frequency of vaginal sex were insignificant (*p* = 0.14 and *p* = 0.23, respectively). However, we found that condom use was significantly associated with the number of sexual partners (*p* = 0.0088). Women with two or more partners reported condom use during vaginal sex more often than women with one partner (81.8% vs. 58.8%). Hence, we then investigated the interaction between the number of sexual partners and condom use and identified significant differences between groups (Figure 2B). However, again, this difference appeared to be predominantly driven by the number of sexual partners.

No significant associations were observed between any intimate hygiene practices and vaginal microbiome beta diversity.

#### 3.3.3 Associations Between Participant Demographics, Intimate Health, Hygiene, and Sexual Practices and Relative Abundance of Vaginal Microbes

**FIGURE 3:**
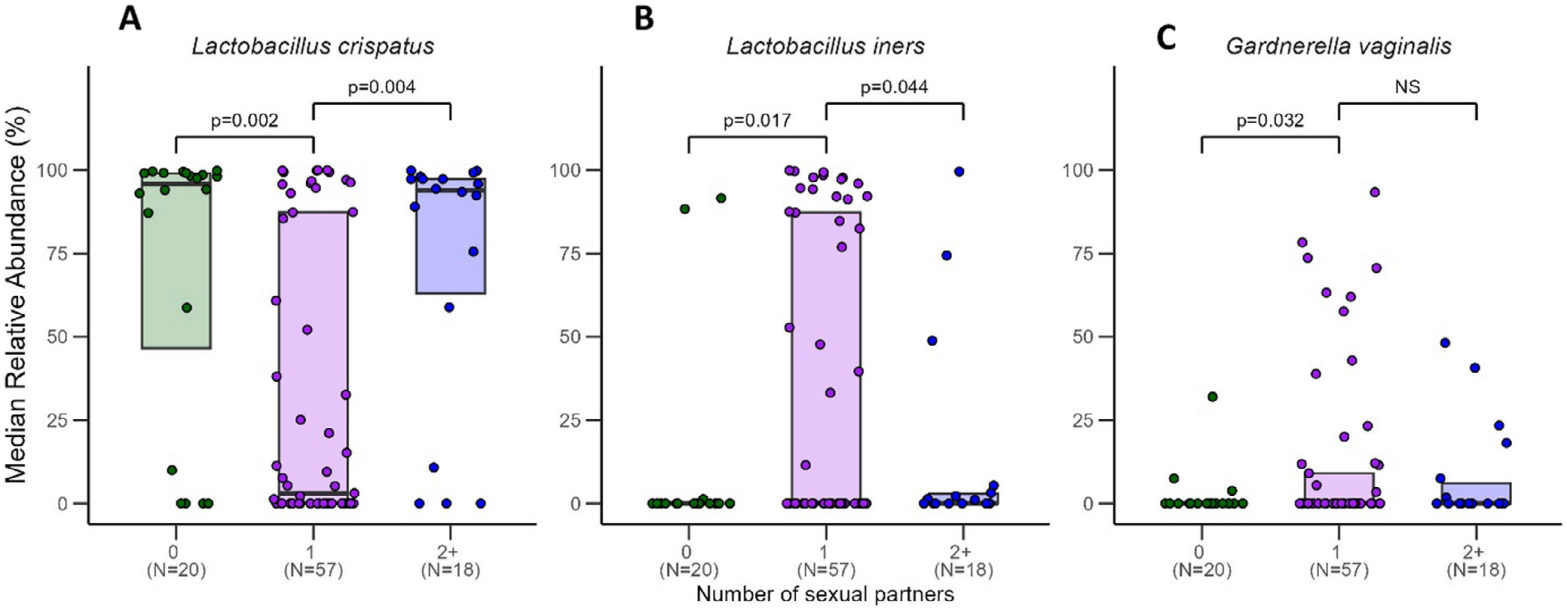
Median relative abundance (%) by number of sexual partner status for three common vaginal species. The plot shows data for (A) *Lactobacillus crispatus* , (B) *Lactobacillus iners* , and (C) *Gardnerella vaginalis* , with statistical significance indicated between conditions. Horizontal lines represent significant differences between groups, with labels indicating *p*‐values for each comparison or “NS” (not significant).

Women who had no sexual partners or two or more sexual partners in the past 6 months had significantly higher relative abundance of *L. crispatus* (*p* = 0.002 and *p* = 0.004, respectively) and lower relative abundance of *L. iners* (*p* = 0.017 and *p* = 0.044, respectively) as compared to women who had one sexual partner in the same time period (Figure 3). Additionally, women who had one sexual partner had higher abundance of *G. vaginalis* than women who had no sexual partners (*p* = 0.032) (Figure 3).

**FIGURE 4:**
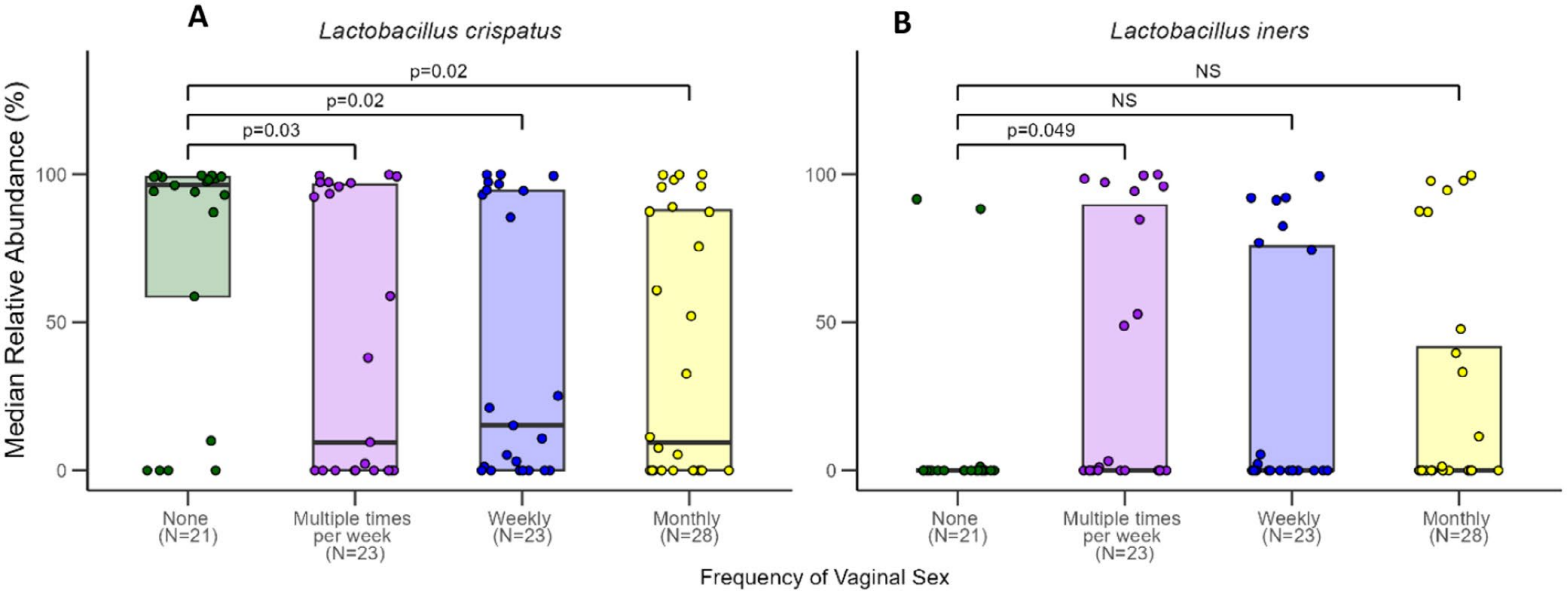
Median relative abundance (%) by frequency of vaginal sex for two common vaginal species. The plot shows data for (A) *Lactobacillus crispatus* and (B) *Lactobacillus iners* , with statistical significance indicated between conditions. Horizontal lines represent significant differences between groups, with labels indicating *p*‐values for each comparison or “NS” (not significant).

Women who were not sexually active in the previous 12 months had significantly higher relative abundance of *L. crispatus* than women who had vaginal sex multiple times per week (*p* = 0.003), weekly (*p* = 0.02), and monthly (*p* = 0.02) (Figure 4A). Additionally, women who had sex multiple times per week had significantly higher abundance of *L. iners* than women who did not have vaginal sex in the previous 12 months (*p* = 0.049; Figure 4B).

**FIGURE 5:**
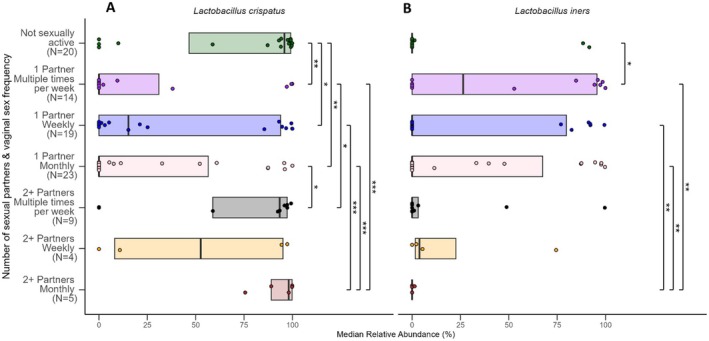
Median relative abundance (%) by number of sexual partners and frequency of vaginal sex for two common vaginal species. The plot shows data for (A) *Lactobacillus crispatus* and (B) *Lactobacillus iners* , with statistical significance indicated between conditions. Horizontal lines represent significant differences between groups, with labels indicating *p*‐values for each comparison (*p < 0.05, **p < 0.01, ***p < 0.001).

**FIGURE 6:**
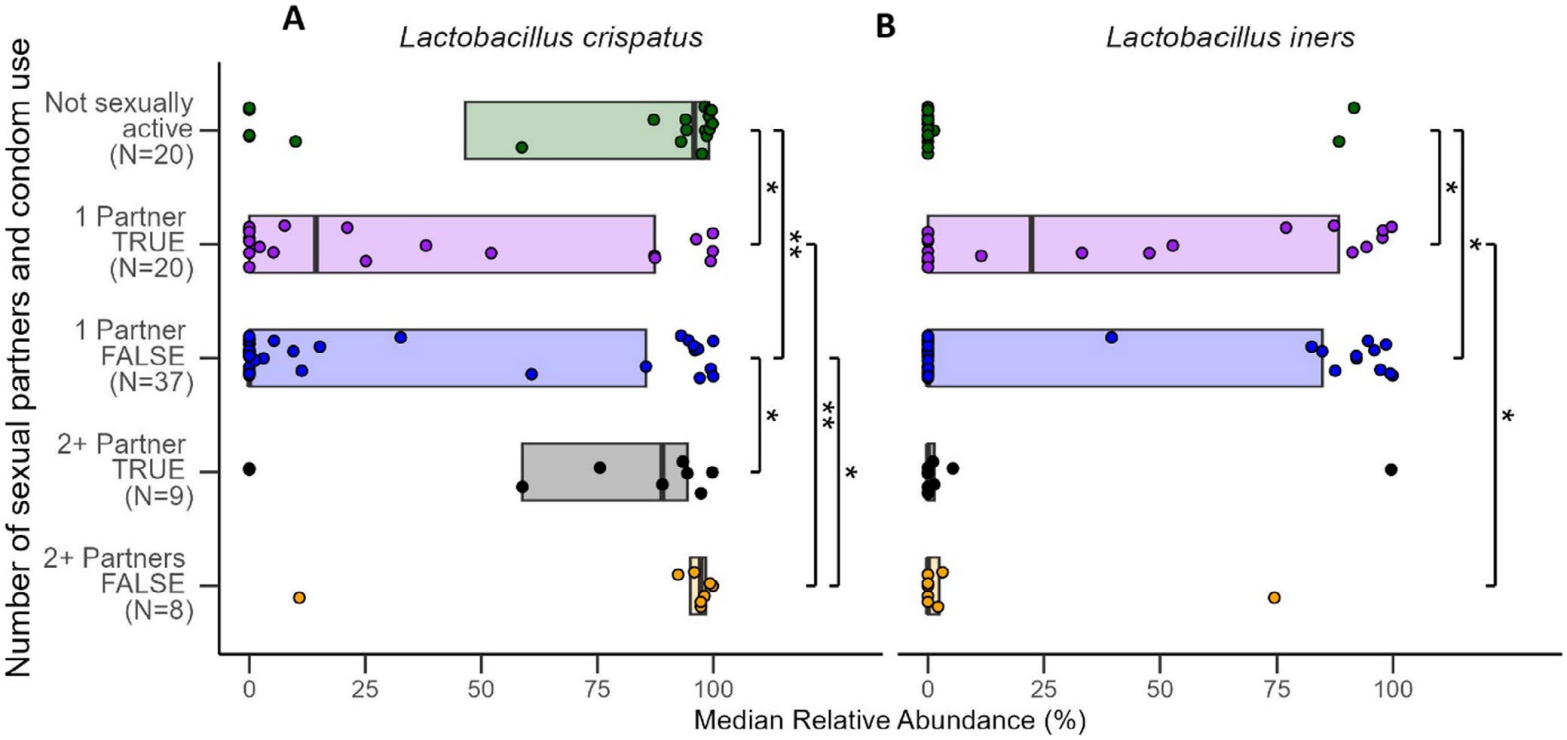
Median relative abundance (%) by number of sexual partners and condom use for two common vaginal species. The plot shows data for (A) *Lactobacillus crispatus* , and (B) *Lactobacillus iners* , with statistical significance indicated between conditions. Horizontal lines represent significant differences between groups, with labels indicating *p*‐values for each comparison (*p < 0.05, **p < 0.01, ***p < 0.001).

To further explore the previously discussed findings in relation to beta diversity among monogamous women, we looked at the interactions between both number of sexual partners/frequency of vaginal sex and number of sexual partners/condom use to determine if there were any particular vaginal microbes that were differentially abundant based on these variables. For both number of sexual partners/frequency of vaginal sex and number of sexual partners/condom use, although there were multiple groups with significantly different levels of *L. crispatus* and *L. iners* , there were no distinct differences observed compared to the individual variables on their own. Hence, monogamous women continued to have lower *L. crispatus* and higher *L. iners* than women with no sexual partners or women with two or more partners (Figures 5 and 6).

**FIGURE 7:**
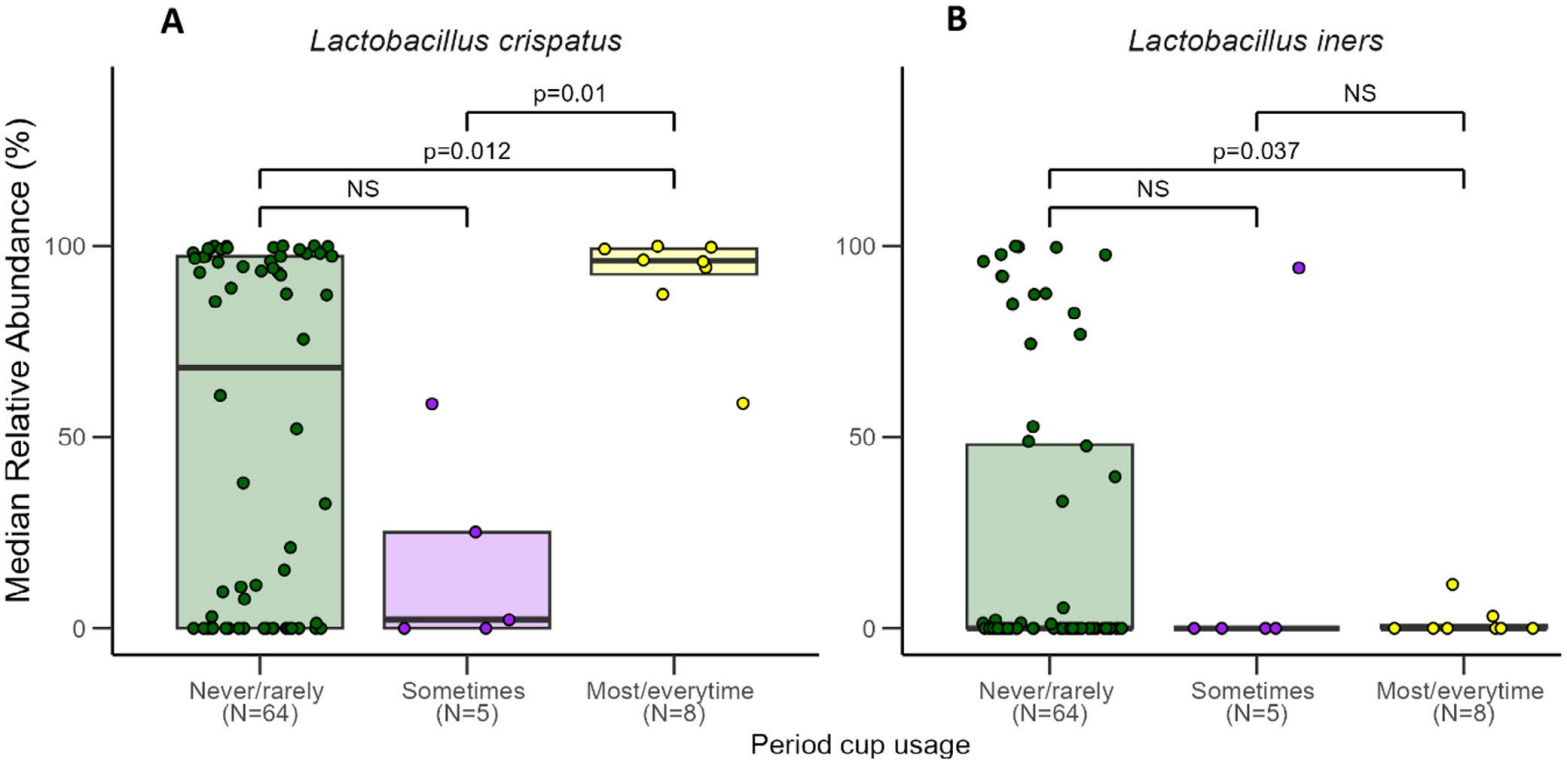
Median relative abundance (%) by use of period cups for two common vaginal species. The plot shows data for (A) *Lactobacillus crispatus* and (B) *Lactobacillus iners* , with statistical significance indicated between conditions. Horizontal lines represent significant differences between groups, with labels indicating *p*‐values for each comparison, or “NS” (not significant).

Using period cups ‘most/every time’ in menstruating women was associated with a higher relative abundance of *L. crispatus* and lower relative abundance of *L. iners* than women who ‘never/rarely’ used period cups (*p* = 0.012 and *p* = 0.037, respectively). Additionally, women who used period cups ‘sometimes’ had lower relative abundance of *L. crispatus* than women who used period cups ‘most/every time’ (*p* = 0.01) (Figure 7).

**FIGURE 8:**
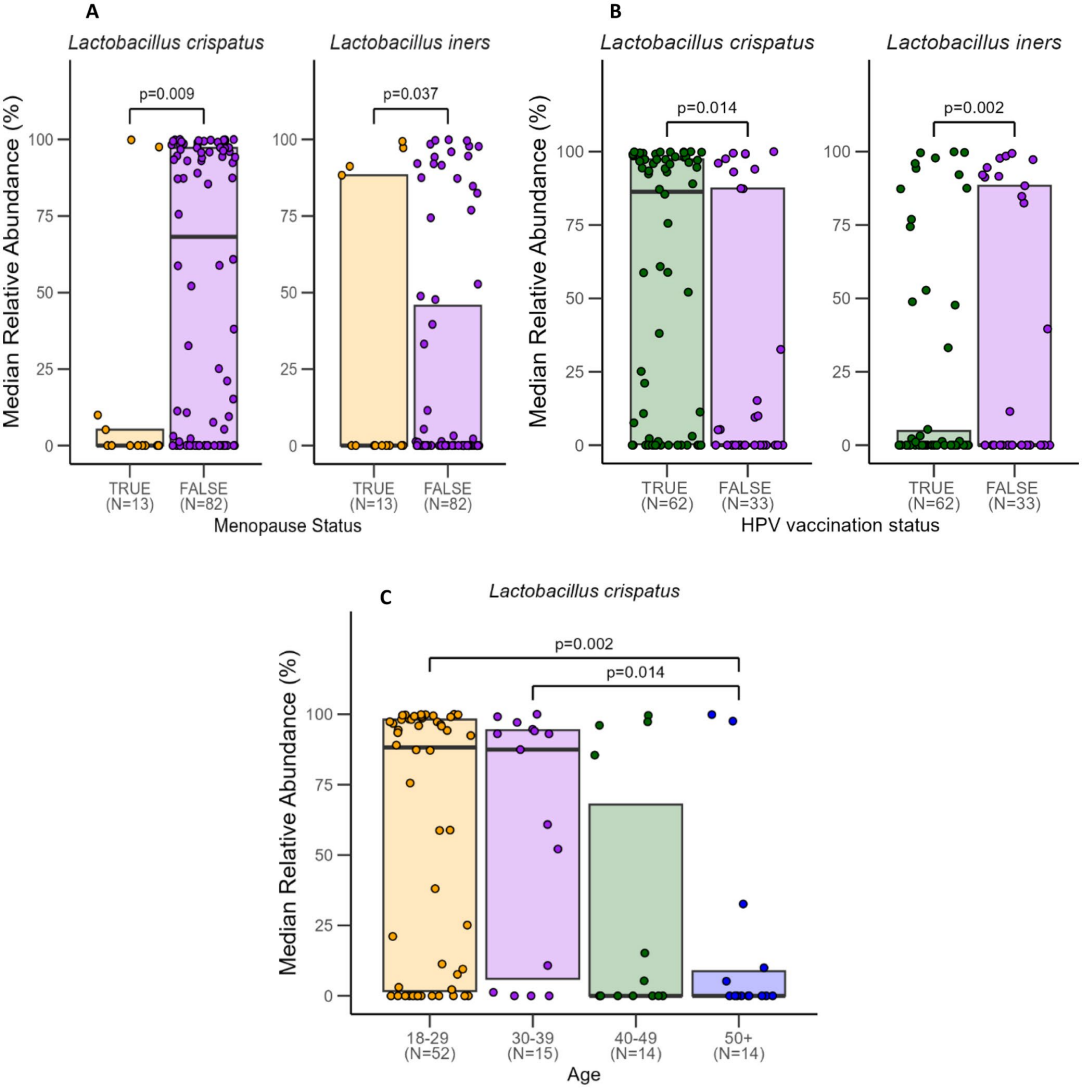
Median relative abundance (%) of common vaginal species between women with respect to (A) menopause, (B) age, and (C) HPV vaccination status. Horizontal lines represent significant differences between groups, with labels indicating *p*‐values for each comparison. One participant who reported menopause in the questionnaire was not included in this analysis due to insufficient amount of sample post‐DNA extraction.

**FIGURE 9:**
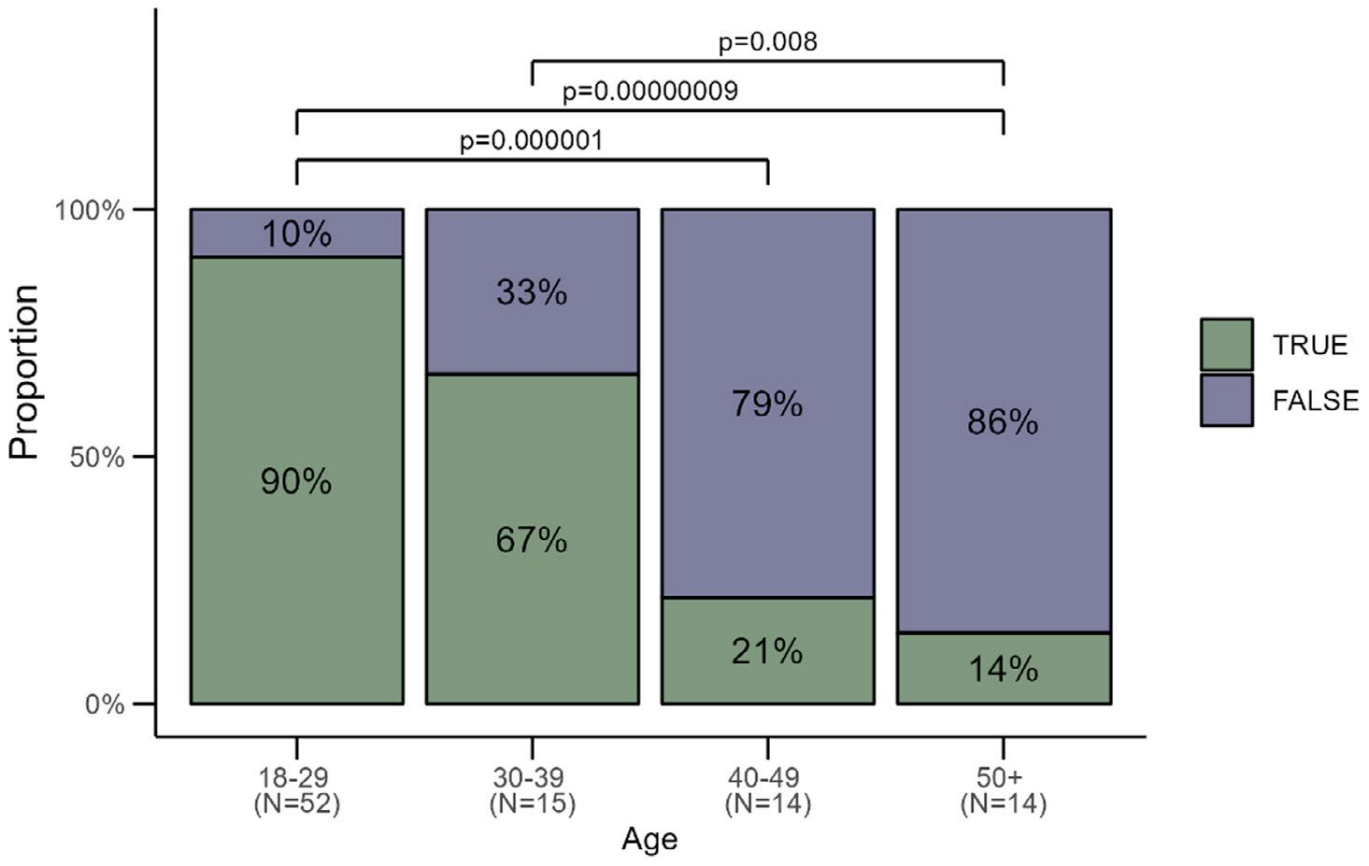
Proportion of women who were vaccinated against HPV across different ages. Women aged 18–29 had significantly higher vaccination rates compared to women aged 40–49 (*p* = 0.00001) and 50+ (*p* = 0.00000009). Similarly, women aged 30–39 had higher vaccination rates than those aged 50+ (*p* = 0.008).

Women who were menopausal had lower relative abundance of *L. crispatus* than women who were not menopausal (*p* = 0.009) can be observed (Figure 8A). It is important to note, however, that two women who were menopausal presented with a high relative abundance of *L. crispatus* , one of which was taking menopause hormone therapy. Figure 8B suggests that women who were aged 50+ years had lower relative abundance of *L. crispatus* than women who were aged 18–29 (*p* = 0.002) and 30–39 years (*p* = 0.014). Although we identified that women vaccinated against HPV had a higher relative abundance of *L. crispatus* and lower relative abundance of *L. iners* than unvaccinated women (*p* = 0.014 and *p* = 0.002 respectively) (Figure 8C), a secondary analysis determined that HPV vaccination status was significantly inversely associated with age (Figure 9).

## 4 Discussion

Utilizing a detailed questionnaire to document the demographics, intimate health, hygiene, and sexual practices among a cohort of non‐pregnant women in Western Australia, several associations were identified with adverse vaginal health conditions. Contrary to our hypotheses, we did not uncover any association between specific intimate hygiene practices and the vaginal microbiome. Instead, it was several lifestyle, sexual, and demographic variables that were found to be associated, with sexual activity, specifically vaginal‐penile sex, exerting the most influence, particularly with respect to the relative abundance of *L. crispatus* and *L. iners*. Monogamous women and women who had regular vaginal sex had lower *L. crispatus* and higher *L. iners* abundance compared to women who had no sexual partners, two or more sexual partners, or had no vaginal sex. All but two women who were not sexually active had a high abundance of *L. crispatus* ; the two women presenting *with L. iners* dominance were aged 50+, with one reporting she was menopausal, and the other describing perimenopausal symptoms, which may explain the *L. iners* dominance [14, 15]. Although we investigated the frequency of vaginal sex and condom use as potential drivers of the observed differences seen in monogamous women, neither was able to explain the distinctly different clustering between the women with a single partner and those either not sexually active or with two or more partners. Our finding that monogamous women tend to have lower *L. crispatus* and higher *L. iners* is especially intriguing; a natural explanation for this would be that frequent sexual intercourse without condom use exposes women to male penile microbiota, within which *L. iners* has been reported on numerous occasions [16, 17]. However, our results instead suggest that such a scenario is not driving the differences observed as neither the frequency of vaginal sex nor condom use could explain this. As the vaginal microbiome is known to be dynamic and impacted by the various menstrual cycle phases, the differences observed could potentially be explained by the time of sampling, in that more women with a single partner may have recently finished menses, after which *L. iners* abundance is known to be elevated [18, 19]. However, as we did not collect highly accurate data on the timing of sample collection, a limitation of most cross‐sectional vaginal microbiome studies, it is difficult to comment on this. Further studies that sample at a universal time point relative to the menstrual cycle are needed to assess the impact of the number of sexual partners on the vaginal microbiome.

*L. crispatus* dominance in this population is promising as it has been described as the most optimal vaginal microbe for its consistent association with positive health outcomes for women including prevention of STI acquisition [20, 21, 22] and improved assisted reproductive technology (ART) outcomes [23]. Low levels of *L. crispatus* have also been associated with preterm birth [24, 25]. Conversely, *L. iners* dominance is associated with a high risk of transition to a diverse, non‐optimal CST IV which is associated with BV, provides less protection against STI acquisition, and is also closely associated with the occurrence of preterm birth [20, 26]. Interestingly, some strain specificity has been observed for *L. iners* , with the ATCC 55195 strain originating from an individual with BV [27, 28] having been reported to have strong pro‐inflammatory properties, while the CCUG 28746T strain isolated from a healthy individual generates little to no pro‐inflammatory response [29]. We did not perform any strain level analyses in this study.

The higher abundance of *L. iners* in monogamous women having frequent vaginal sex may suggest the microbe to be sexually transmitted, or to at least favor the conditions post‐sexual intercourse in which the vaginal microbiome may be disrupted due to the introduction of the penis and/or seminal fluid. The optimal penile microbiome is low‐diversity and *Corynebacterium*‐dominant with regard to the reproductive health of men and their female partners [17]; however, *L. iners* has been identified in the male reproductive tract microbiome on numerous occasions [16, 17, 30, 31]. Semen, an alkaline fluid contrasting the acidic vaginal microenvironment, also harbors its own diverse microbiome [31, 32]. Mändar, Punab [32] investigated heterosexual couples seeking in vitro fertilization (IVF) treatment and found that there was an 85% similarity between microbes across the seminal and vaginal microenvironments, despite pH differences. In addition, they found a decrease in *L. crispatus* in the vagina post‐sexual intercourse [32], supporting our findings. Dixon et al. [33] showed multiple cases of sexual transmission of microbes between couples, with the number of amplicon sequence variants present in vaginal swabs from female partners increasing post‐intercourse, while the number in male partners' penile swabs decreased. However, the sexual transmission study identified low abundance of *L. iners* in the male and high abundance in the female before intercourse, and the opposite after intercourse, suggesting that in this case, *L. iners* was likely sexually transmitted from the female to the male partner [33]. Recently, Vodstrcil et al. [34] demonstrated that male‐partner treatment for BV reduced recurrence in female partners by 63%. The effectiveness of this concurrent partner treatment for BV is key evidence that exposure to the penis during sex impacts the vaginal microbiome. Interestingly, we did not observe any difference in women who used condoms compared to those who did not.

In our study, menopausal women were most likely to cluster into CST IV, least likely to cluster into CST I, and had lower relative abundance of *L. crispatus* . These findings are consistent with previous studies [35] including Brotman, Shardell [14] who found that perimenopausal women were more likely to be CST I or CST III whereas menopausal women were more likely to be CST IV. High estrogen levels are associated with increased free glycogen in vaginal epithelial cells which acts as a food source for *Lactobacillus* spp. [15]. Decreasing levels of circulating estrogen at menopause are known to cause a depletion in *Lactobacillus* spp. [36, 37, 38]. Surprisingly, we did not find an association between hormonal or barrier contraception and the vaginal microbiome, despite an association being well established [16, 39, 40]. Brooks, Edwards [39] for example, demonstrated that hormonal contraceptive users (*n* = 682) were more likely to have high *Lactobacillus* spp. titres. Additionally, hormonal contraceptives such as the combined oral contraceptive pill (COCP) have been linked to a reduced BV incidence [41, 42, 43].

Whilst HPV vaccination status in our study was significantly associated with higher relative abundance of *L. crispatus*, we also documented a strong inverse association between age and HPV vaccination status itself. This was not surprising considering that the National immunization programme for HPV was implemented in 2007 for girls in Australia; hence, women aged 18–29 years in 2022 would have all had the option of vaccination during high school [44]. In comparison, older women in our cohort may not have had ready access to such a programme and therefore remained unvaccinated. As age itself was also associated with higher *L. crispatus* relative abundance, it is difficult to untangle which specific factor is driving this observation or, alternatively, if it is a combination of both factors. However, there is some evidence that HPV vaccination itself does not impact the vaginal microbiome, with Giraldo et al. [45] identifying no changes in vaginal microbial composition between pre‐vaccination state and post‐third dosage. HPV infection, however, has been well‐established as associated with *Lactobacillus* spp.‐depleted vaginal microbial states [46, 47, 48, 49].

Most women in our study reported washing externally around the vulvar area (86.7%) with water only, soap +/− water, or shower gels. Water only was the most popular cleansing method used by 86.2% of external cleansers. We observed that 20.5% of women reported using shower gel daily to cleanse externally, even though these products are not designed for the intimate area and are often heavily scented. The Royal College of Obstetricians and Gynecologists generally advises against using water combined with shower gels for vulval cleansing as they can dry and irritate the skin, especially for those with vulval skin disorders [10]. Their recommendation is to use water with a small amount of soap substitute on the vulva only once per day. Pubic grooming was also standard in our cohort, practised by 89.6% of participants. Removal of pubic hair is common for aesthetic reasons [7] and appears more popular among younger women. Erekson et al. [50] conducted an intimate hygiene survey of postmenopausal women and found that most (78.1%) did not report pubic grooming. Our study's high proportion of pubic grooming may therefore reflect our younger demographic. It has been hypothesized that pubic grooming causes skin microtrauma and may contribute to the potential for infection in the vulvovaginal area [1, 51]. However, we did not observe any significant relationships between pubic grooming, vaginal infections or symptoms, and the vaginal microbiome.

Whilst no women in our cohort reported douching, 17.7% practised internal (vaginal) washing. This is lower than the estimated one third of US women who practice vaginal douching [8]. In Canada, Crann, Cunningham [12] reported internal washing by 64% and vaginal douching by 21.3% of their participants. Differences in reported rates of internal washing may reflect questionnaire design. Crann, Cunningham [12] inquired about the lifetime use and regular use of hygiene products; whilst we asked about the current, regular use of hygiene products most likely to influence the microbiome at the time of vaginal swab collection. We also had a significantly smaller sample size (*n* = 95 vs. Crann, Cunningham [12] *n* = 1435) and did not capture the hygiene practices of any women of African ethnicity, who are known to douche more frequently [8, 10].

We observed a trend towards internal washing and increased rates of adverse vaginal symptoms and infections consistent with previous douching studies [8, 12, 52, 53]. Specifically, we discovered recurrent thrush to be more common among women who washed internally. Vaginal cleansing is known to cause *Lactobacillus* spp. depletion in the vagina [8, 54], which increases opportunistic colonization of *Candida* spp., predisposing women to thrush. Although consistent with previous studies [8, 54] it remains unclear whether internal washing causes recurrent thrush or whether these women are more likely to wash internally to alleviate thrush symptoms, the most common of which is an intense itch. An analysis of motivations for internal washing behaviors in women suffering from recurrent vaginal infections may help to shed light on this issue.

We also demonstrated a link between regular perineal washing and reduced vaginal infections. The perineum refers to the area of skin separating the vagina from the anus and is the most likely pathway of transmission for gastrointestinal bacteria such as *Escherichia coli* and Group B Streptococcus into the vagina [55, 56]. *E. coli* is the most common organism causing urinary tract infections (UTIs) in women [57, 58]. It is estimated that 25% of women will experience a UTI recurrence within 6 months of the initial infection, and this is often due to periurethral contamination [55]. Recurrent UTIs were also prevalent in our cohort, with 17 participants reporting two or more UTIs in the past year. Given the high risk of recurrence, the introduction of regular perineal washing into a woman's intimate hygiene routine may help reduce the risk of vaginal infections. Future studies assessing a perineal washing intervention on a cohort of women with recurrent UTIs would be helpful to determine its potential clinical relevance.

Previous studies have suggested that CSTs are dynamic and change throughout the menstrual cycle. *L. iners* and anaerobes such as *G. vaginalis* appear most abundant during menses and the follicular phase but decline later in the menstrual cycle when *L. crispatus* predominates [18, 19]. For our study, women with regular periods were grouped based on the time of sampling into the follicular, ovulatory, or luteal phases of the menstrual cycle based on the number of days since their last period. Most women were in the luteal phase (53.3%), which is characterized by high progesterone and estrogen levels. No associations between menstrual phases, relative abundance of *L. crispatus* , and CSTs were identified. Frequency counts for each menstrual phase were low; therefore, it would be beneficial for this relationship to be re‐examined in a larger cohort of women with vaginal sampling at multiple points throughout the menstrual cycle.

Our study demonstrated an association between *L. crispatus* and *L. iners* abundance and use of period cups; however, our sample size of women using this product was very small and therefore the results may be inflated. Notably, there was a high rate of tampon usage in our cohort. This is consistent with previous reports on Australia and other high‐income countries, especially in young women, and should be considered when interpreting our findings [59, 60]. Studies have found that intravaginal menstrual products may impact the vaginal microbiome; however, it is extremely difficult to differentiate the changes that may be induced by menstrual product use and those that are due to menses itself [60]. Further research into period products and their impact on the vaginal microbiome is needed to confirm this association. We hypothesized that heavy menstrual flow would be associated with increased abundance of *L. iners* (CST III) as it is most abundant during menses and appears to grow better in anaerobic conditions on blood agar [18, 19, 61]. However, no associations between heavy menstrual flow and CST were found.

Although ethnicity is believed to be a driver of women having CST IV microbiomes, our cohort was 93% Caucasian; hence, due to low numbers, a statistical analysis regarding the impact of ethnicity on microbial profiles was not feasible. However, 4 of the 6 non‐Caucasian participants (66.6%) were CST I, and the remaining two were CST III. Hence, all women in CST IV (*n* = 16) were Caucasian, and therefore, it is most likely that this was a ‘non‐optimal’ state for these women [4]. However, we did not find any association between CST assignment and adverse vaginal symptoms or conditions. This reinforces the nuance of characterizing CST IV as ‘non‐optimal’, as we would have expected to observe higher rates of adverse vaginal outcomes in these participants [4].

Our study has several strengths. We were the first to use a questionnaire combined with a microbial DNA analysis of mid‐vaginal samples to gain insight into the intimate health and hygiene practices and their associated impacts on the vaginal microbiome. In contrast to previous studies which were vague in their definition of ‘vaginal washing’, [11] we were able to accurately distinguish internal from external washing by defining each respective area and incorporating highlighted diagrams into questions. We also sequenced the full‐length 16S rRNA gene in the vaginal microbiome, which enabled assessment of the relative abundance of as many bacterial species as possible, reduced bias, and improved the sensitivity and specificity of taxonomic profiling.

Limitations of our study include our small sample size (*n* = 96), which resulted in the low frequency of several intimate hygiene practices and vaginal symptoms/conditions. Our samples were also self‐collected. Although previous studies have shown concordance between self‐collected and physician‐collected vaginal swabs, there is still some potential for individual variation, such as contamination from the external genital area, which could potentially influence the microbial composition observed [62]. We also recruited many university‐educated women who may be biased towards certain intimate hygiene practices that are not reflective of the broader Australian population. However, this provides data for a relatively homogenous Caucasian population of women aged between 18 and 29 years in a high‐income country. Our survey also had minor technical flaws, including a participant's ability to give more than one answer to several frequency questions. Additionally, we did not account for smoking status or capture the use of sex toys in women who were not sexually active. There was a risk of recall bias when completing the survey as participants were asked about the frequency of use of feminine hygiene products and sexual activity and the number of days since their last period. Additionally, recall error in calculating the days since their last menstruation could have resulted in participants being placed into the wrong menstrual phase. However, our approach allowed us to include as many participants as possible in phase‐specific analyses while accounting for variability and uncertainty in self‐reported menstrual timing.

This study is the first of its kind to capture the intimate hygiene practices of Australian women and use full‐length 16S rRNA gene sequencing to analyze their vaginal bacterial communities. Finally, and perhaps most importantly, our results serve as a foundation for future vaginal microbiome research, especially with a focus on sexual activity and understanding the dynamic between heterosexual partners. Women have historically been solely responsible for the treatment of adverse vaginal conditions without consideration of the impact of their male partners. This conversation is beginning to shift, and the results of this study continue that momentum.

## Disclosure

The authors have no relevant financial, personal, political, or religious interests that could have influenced the work reported in this manuscript.

## Ethics Statement

This study was conducted in accordance with the ethical standards of the University of Western Australia Human Research Ethics Committee (2022/ET000242).

## Consent

All participants were required to provide written, informed consent.

## Conflicts of Interest

The authors declare no conflicts of interest.

## Supporting information


**Table S1:** Raw read counts of species present in extraction and PCR controls.

**Appendix S1:** Feminine health and hygiene practices survey.

## Data Availability

The data that support the findings of this study are openly available in NCBI at https://aus01.safelinks.protection.outlook.com/?url=http%3A%2F%2Fwww.ncbi.nlm.nih.gov%2Fbioproject%2F1293858&data=05%7C02%7C22734868%40student.uwa.edu.au%7C3ac2b4d9d34c40eab6db08ddc810afcd%7C05894af0cb2846d8871674cdb46e2226%7C0%7C0%7C638886696248371566%, reference number PRJNA1293858.
