# Supplementary material for: Investigating the Link Between Intimate Health, Hygiene and Sexual Practices and the Vaginal Microbiome—The INTIMATE Study
Source: Reprod Med Biol. 2025 Oct 21;24(1):e12685. doi: 10.1002/rmb2.12685 (PMC12538639; doi:10.1002/rmb2.12685)
Supplement: Supplementary file 1 — Table S1: Raw read counts of species present in extraction and PCR controls. [file RMB2-24-e12685-s001.docx]

**Supplementary Table 1: Raw read counts of species present in extraction and PCR controls**

|  | EXT_CONT_1 | EXT_CONT_2 | EXT_CONT_3 | EXT_CONT_4 | PCR_NEG_1 | PCR_NEG_2 | PCR_NEG_4 | PCR_NEG_5 |
| --- | --- | --- | --- | --- | --- | --- | --- | --- |
| *Lactobacillus crispatus* | 0 | 0 | 0 | 9 | 16 | 8 | 0 | 2 |
| *Lactobacillus iners* | 1 | 0 | 1 | 18 | 3 | 1 | 0 | 2 |
| *Gardnerella vaginalis* | 0 | 0 | 0 | 3 | 2 | 0 | 1 | 0 |
| *Lactobacillus mulieris* | 0 | 0 | 0 | 4 | 0 | 0 | 0 | 0 |
| *Ureaplasma parvum* | 0 | 0 | 0 | 2 | 0 | 0 | 0 | 0 |
| *Campylobacter ureolyticus* | 0 | 0 | 0 | 2 | 0 | 0 | 0 | 0 |
| *Bifidobacterium longum* | 0 | 0 | 0 | 12 | 0 | 0 | 1 | 0 |
| *Lacticaseibacillus paracasei* | 0 | 0 | 0 | 4 | 0 | 0 | 0 | 0 |
| *Frigoribacterium sp*. | 32 | 0 | 0 | 0 | 0 | 0 | 0 | 0 |
| *Pelomonas sp*. | 28 | 0 | 0 | 0 | 0 | 0 | 0 | 0 |
