# Supplementary figures and images for: Investigating the Link Between Intimate Health, Hygiene and Sexual Practices and the Vaginal Microbiome—The INTIMATE Study
Source: Reprod Med Biol. 2025 Oct 21;24(1):e12685. doi: 10.1002/rmb2.12685 (PMC12538639; doi:10.1002/rmb2.12685)

##
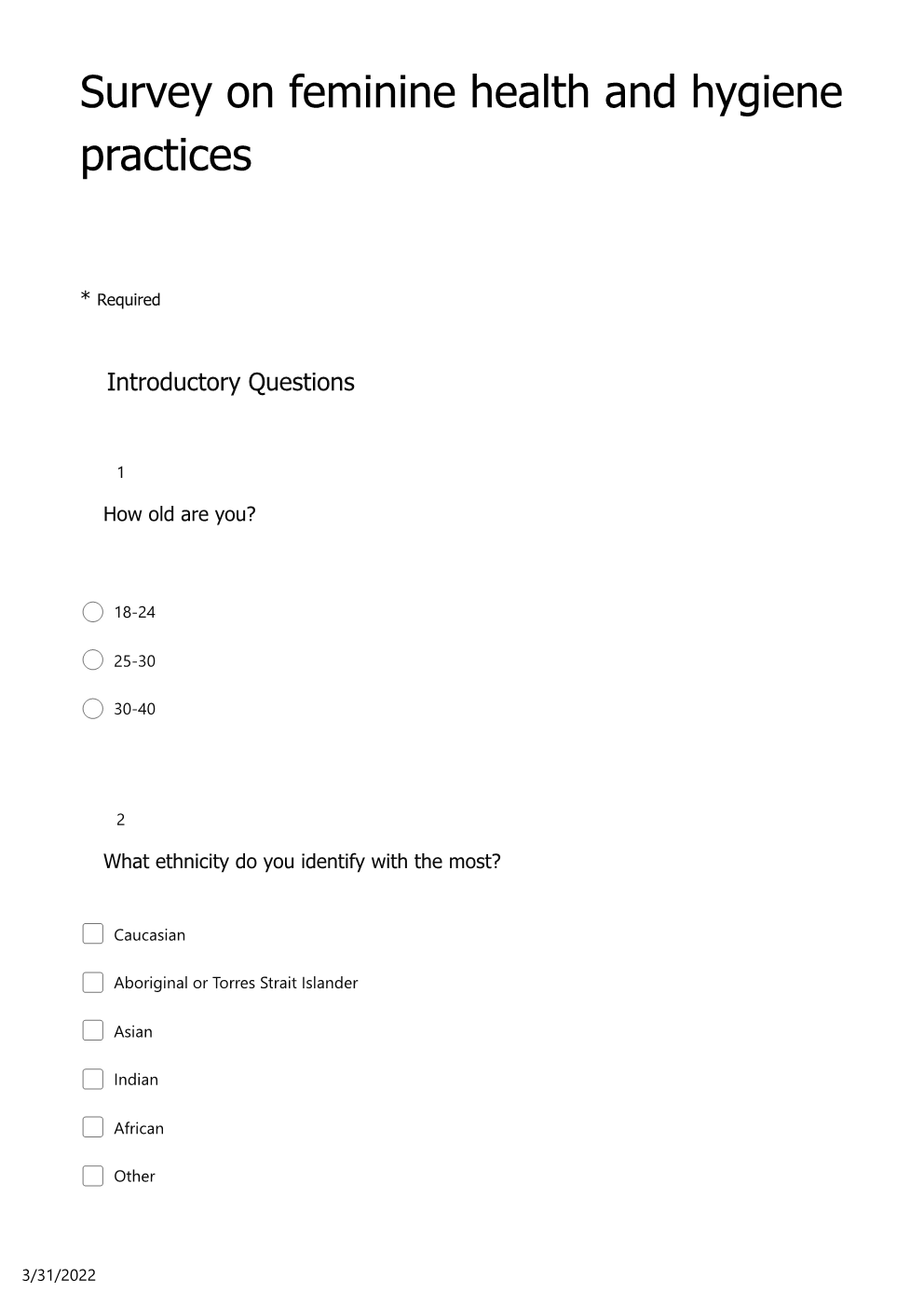
Appendix 1:

**
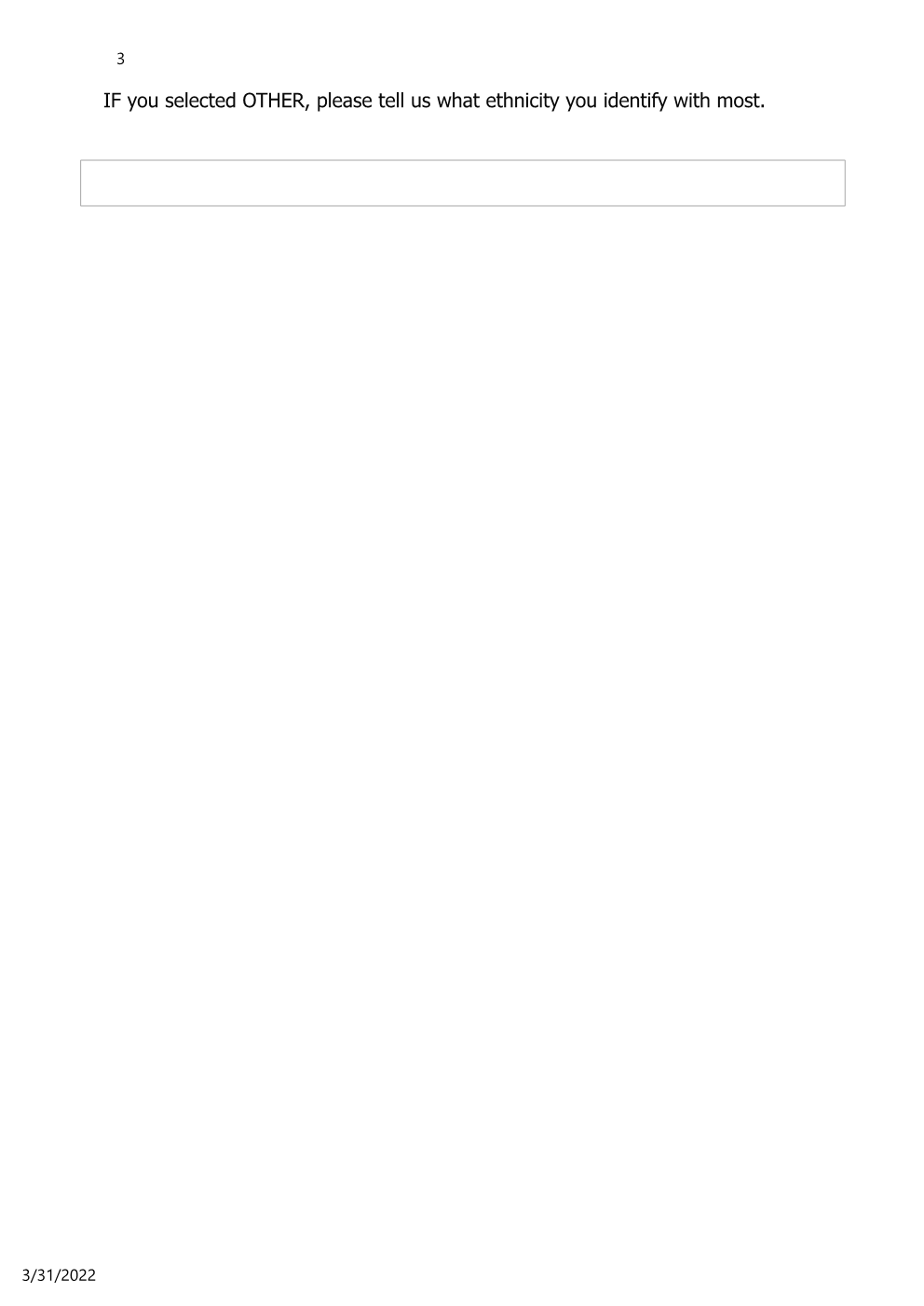

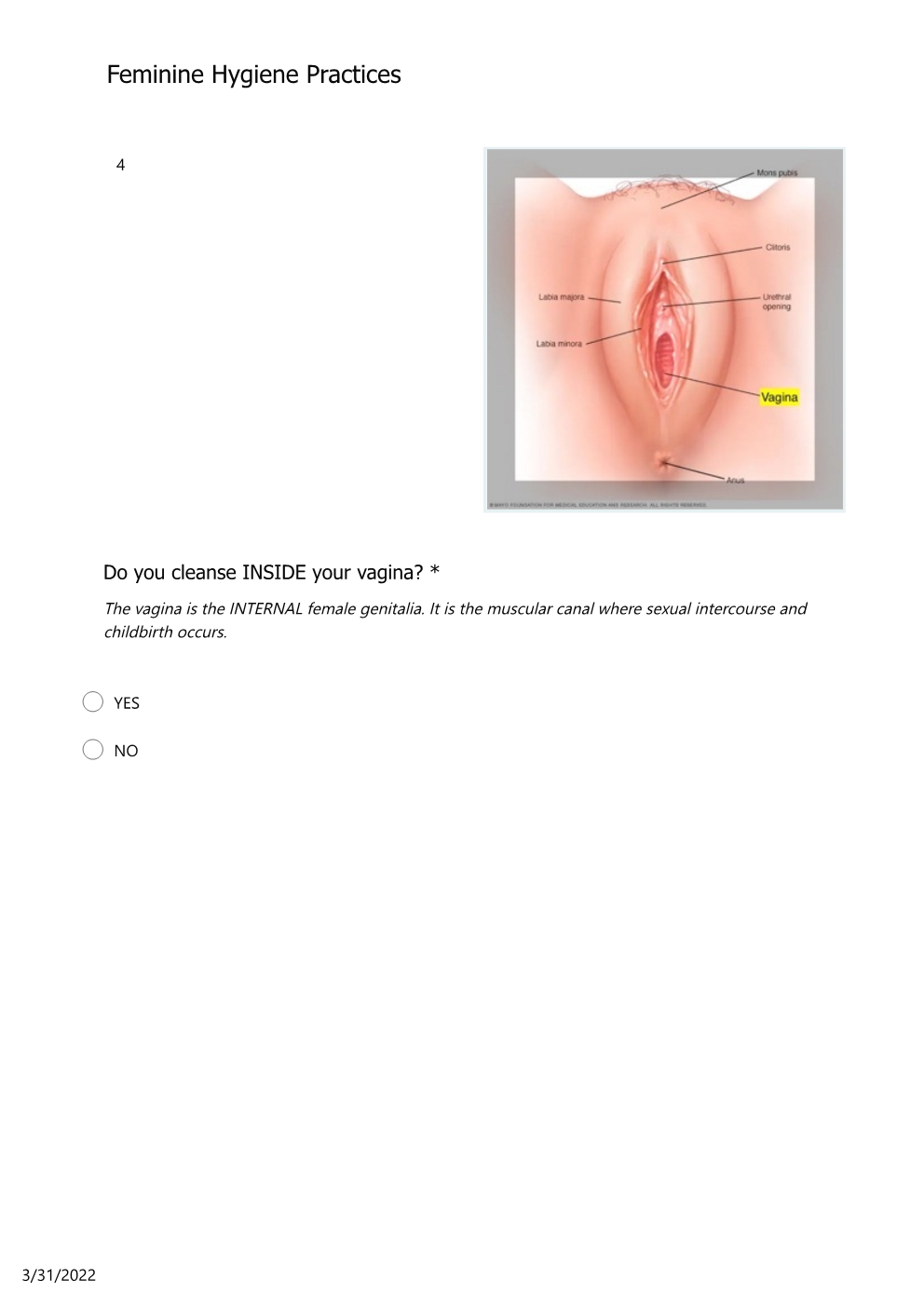

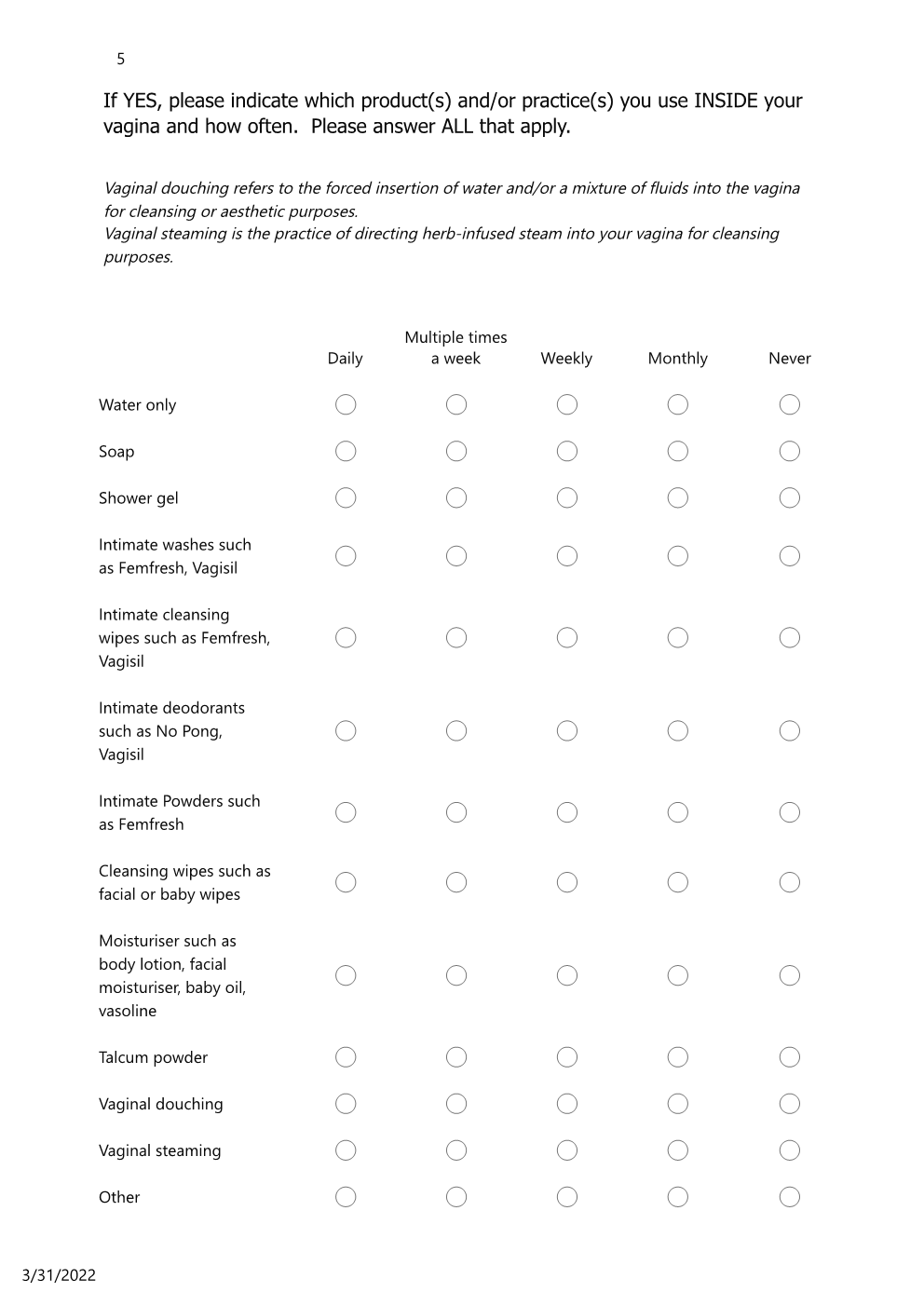

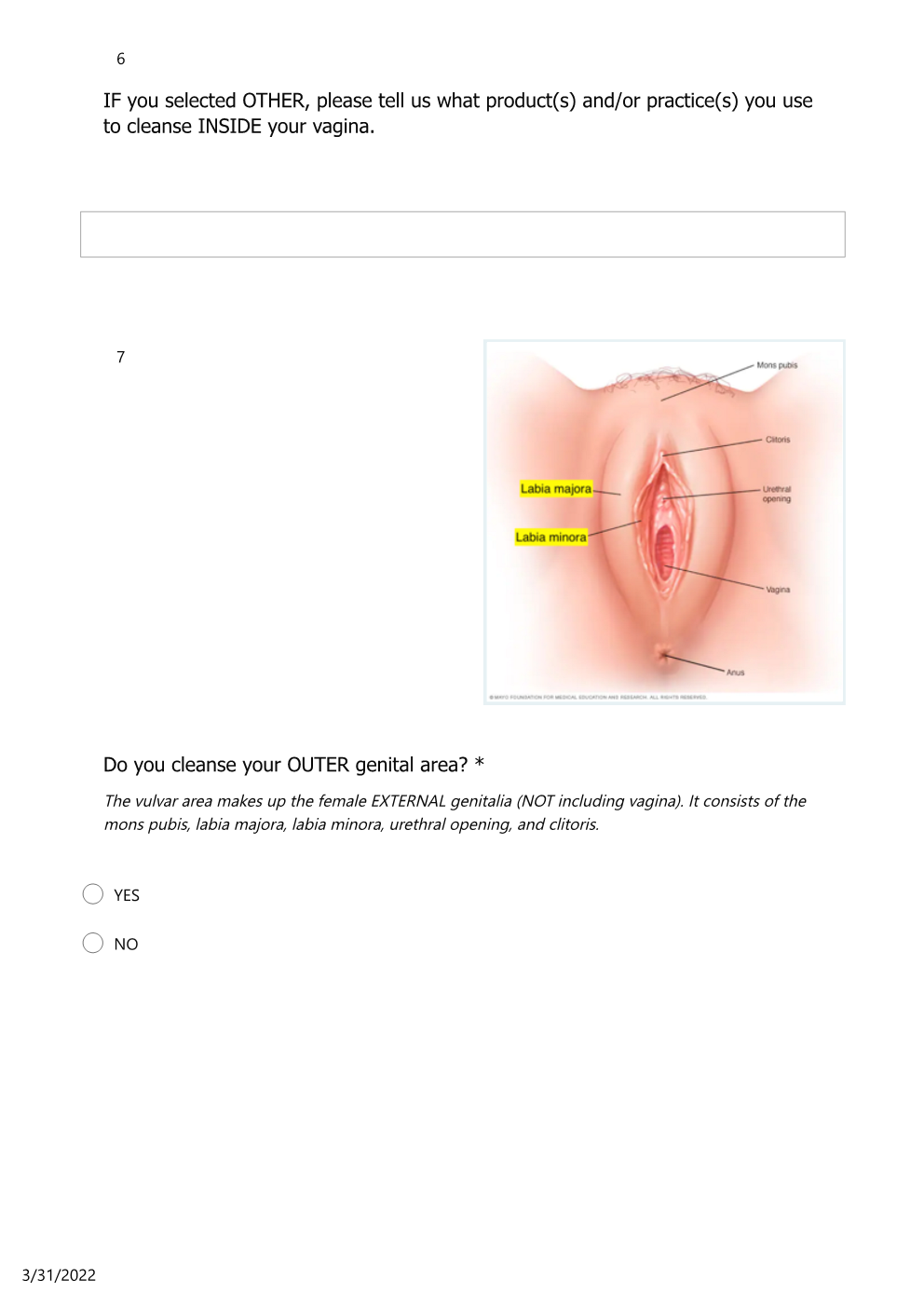

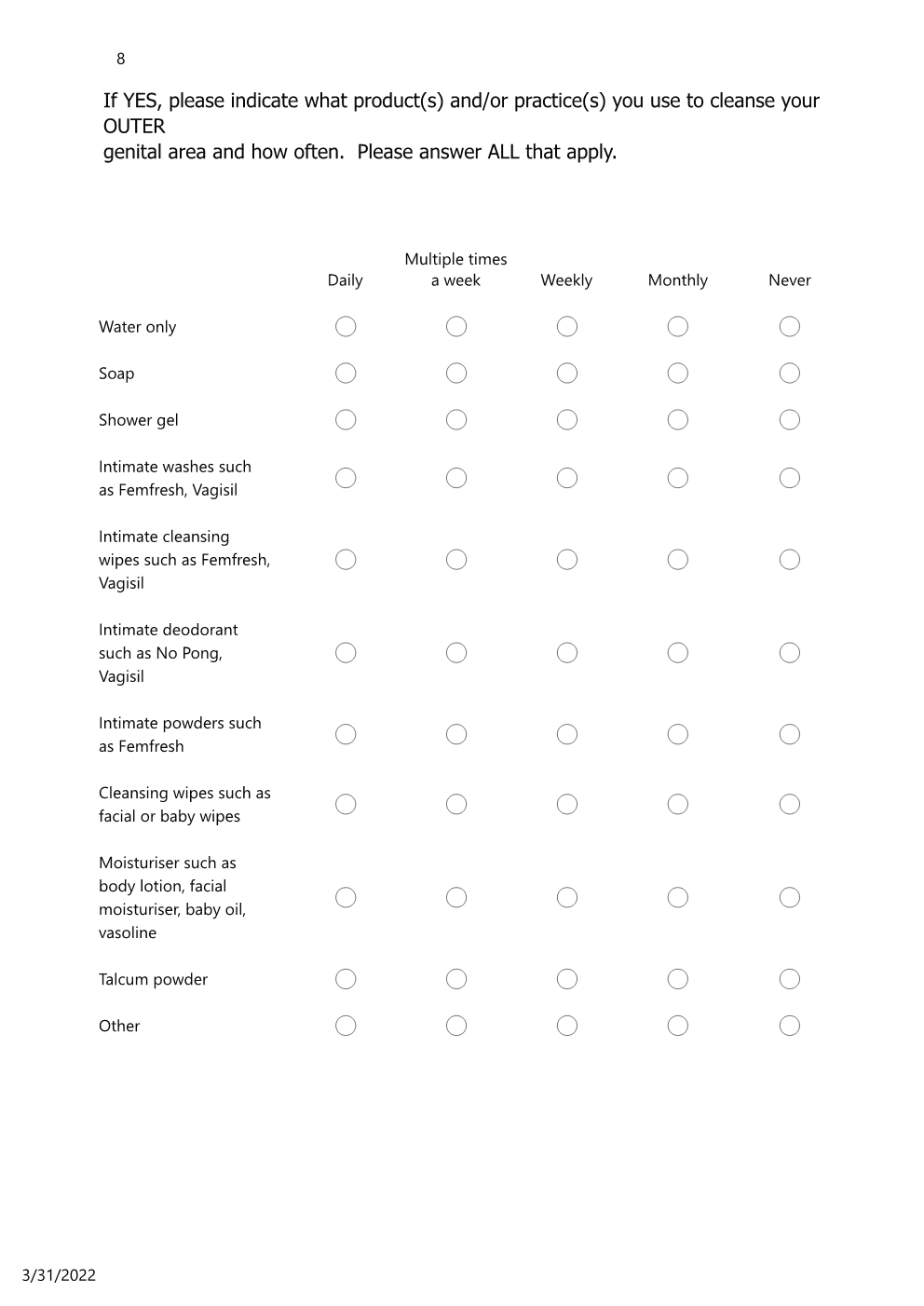

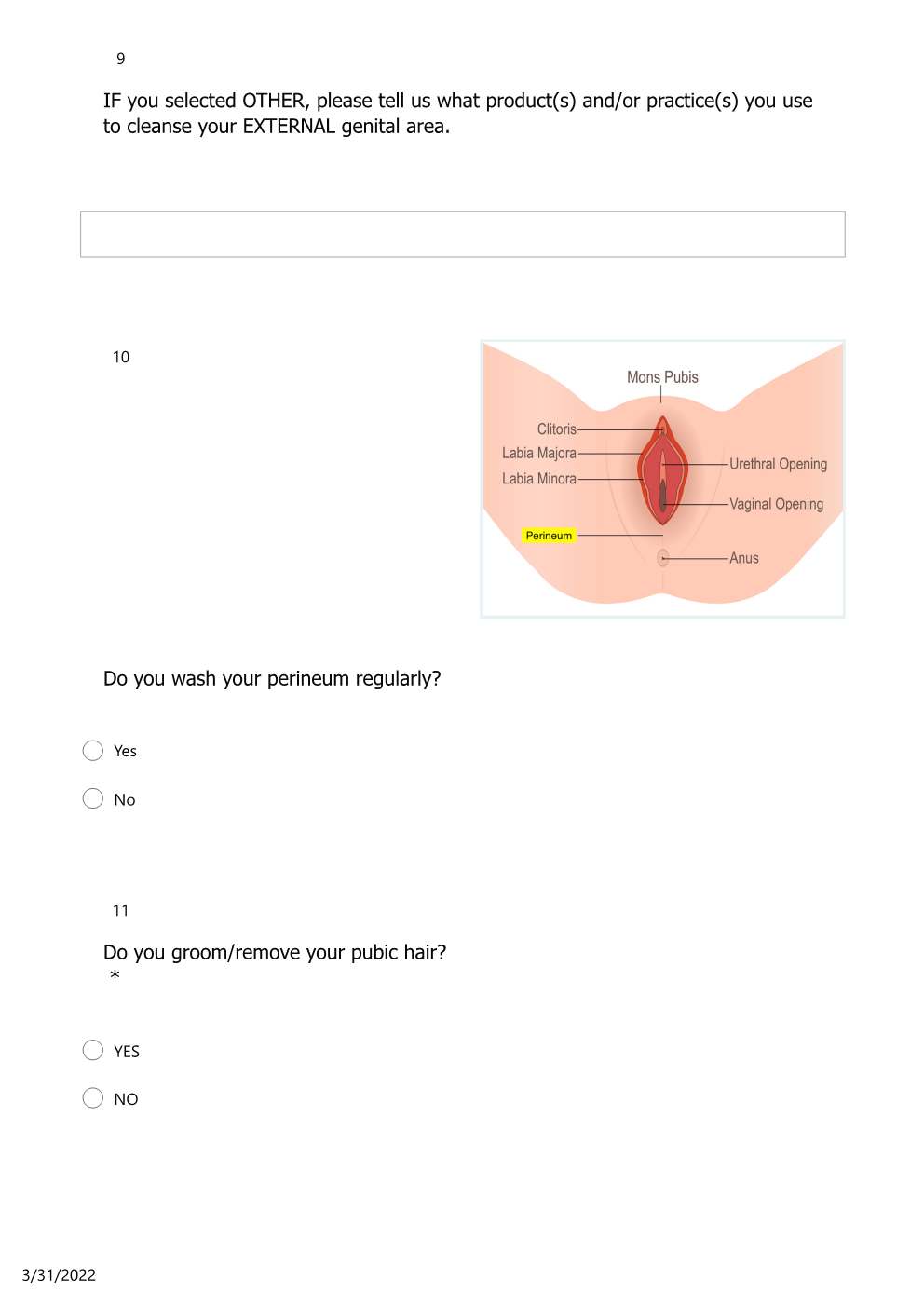

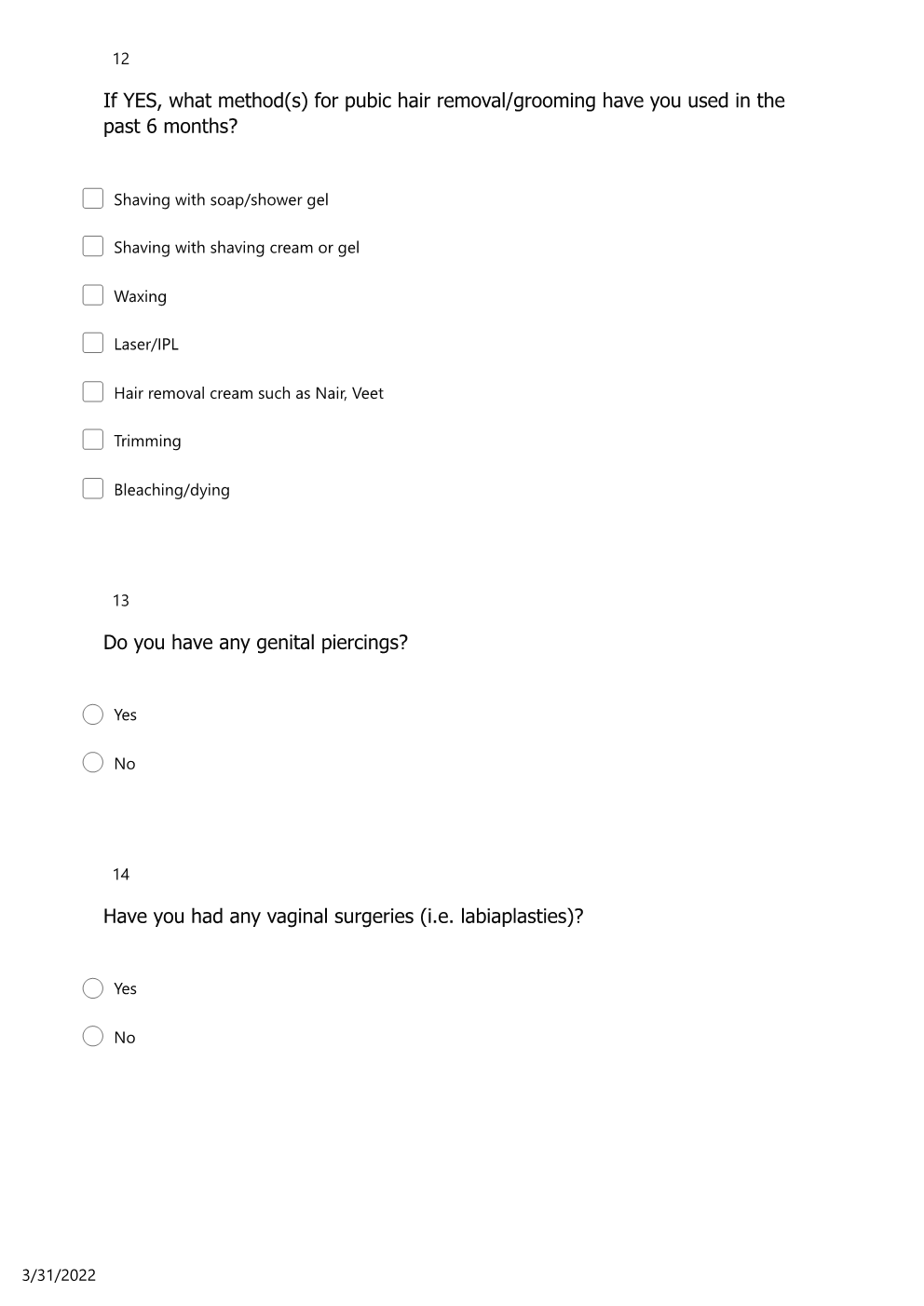

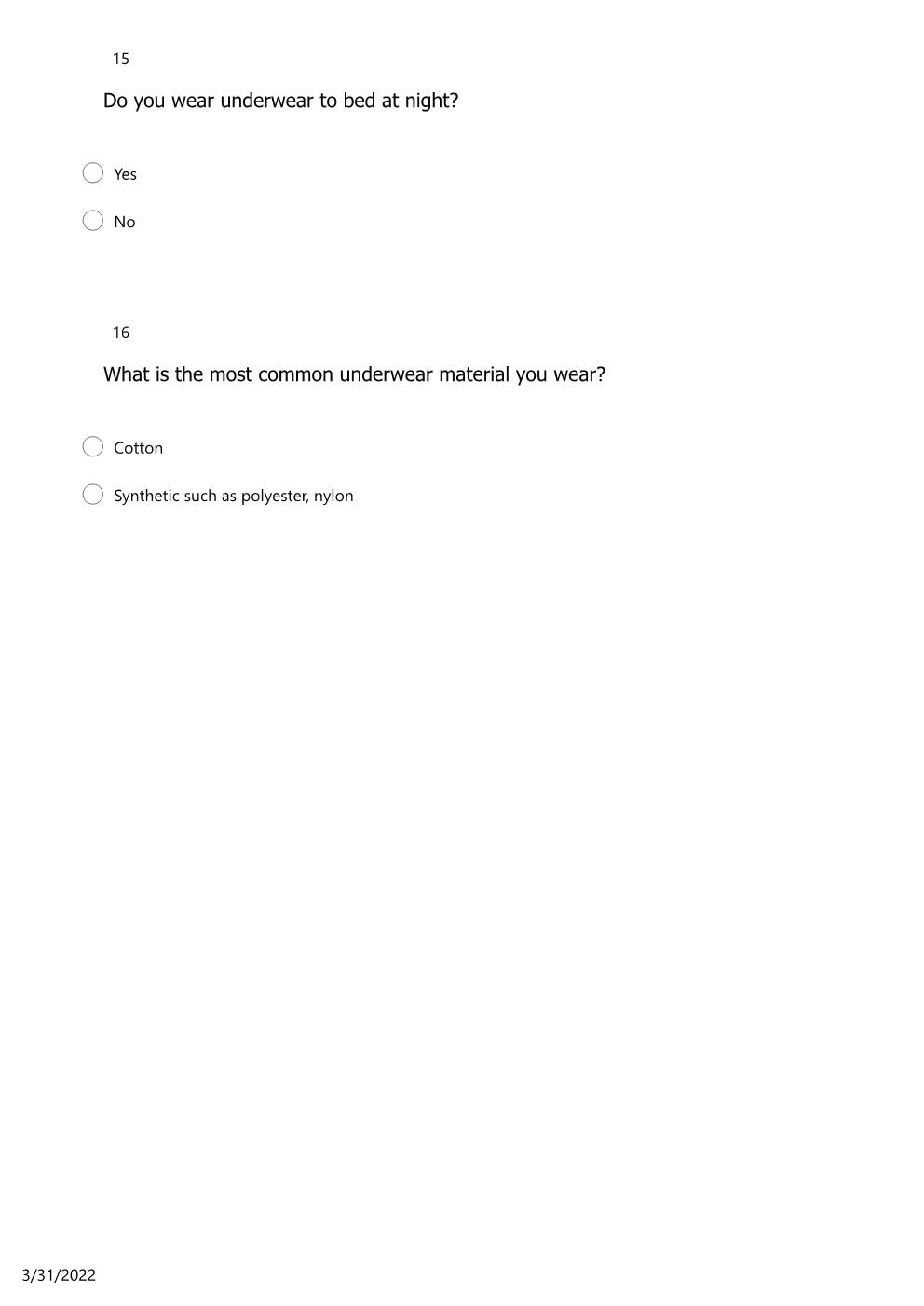

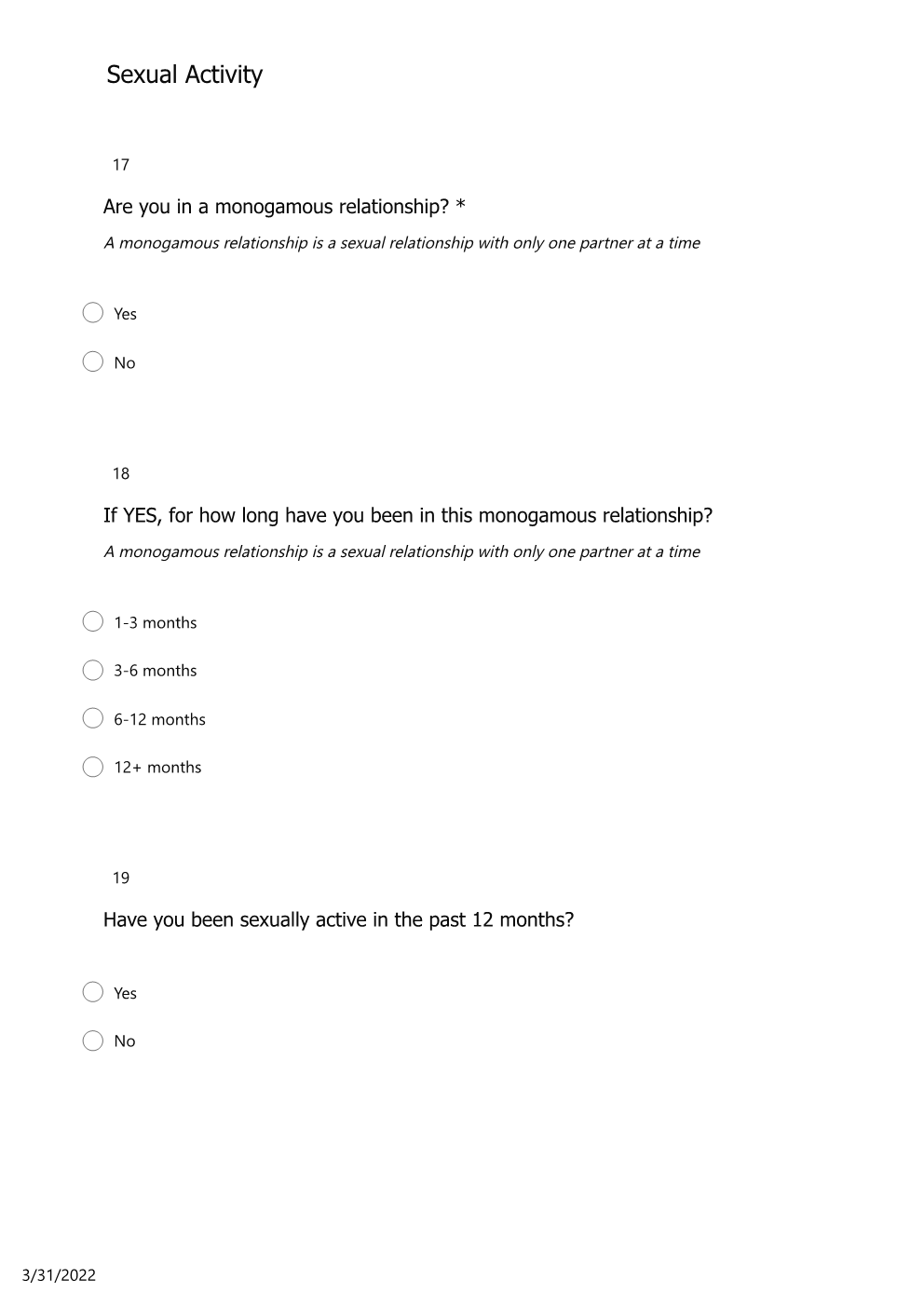

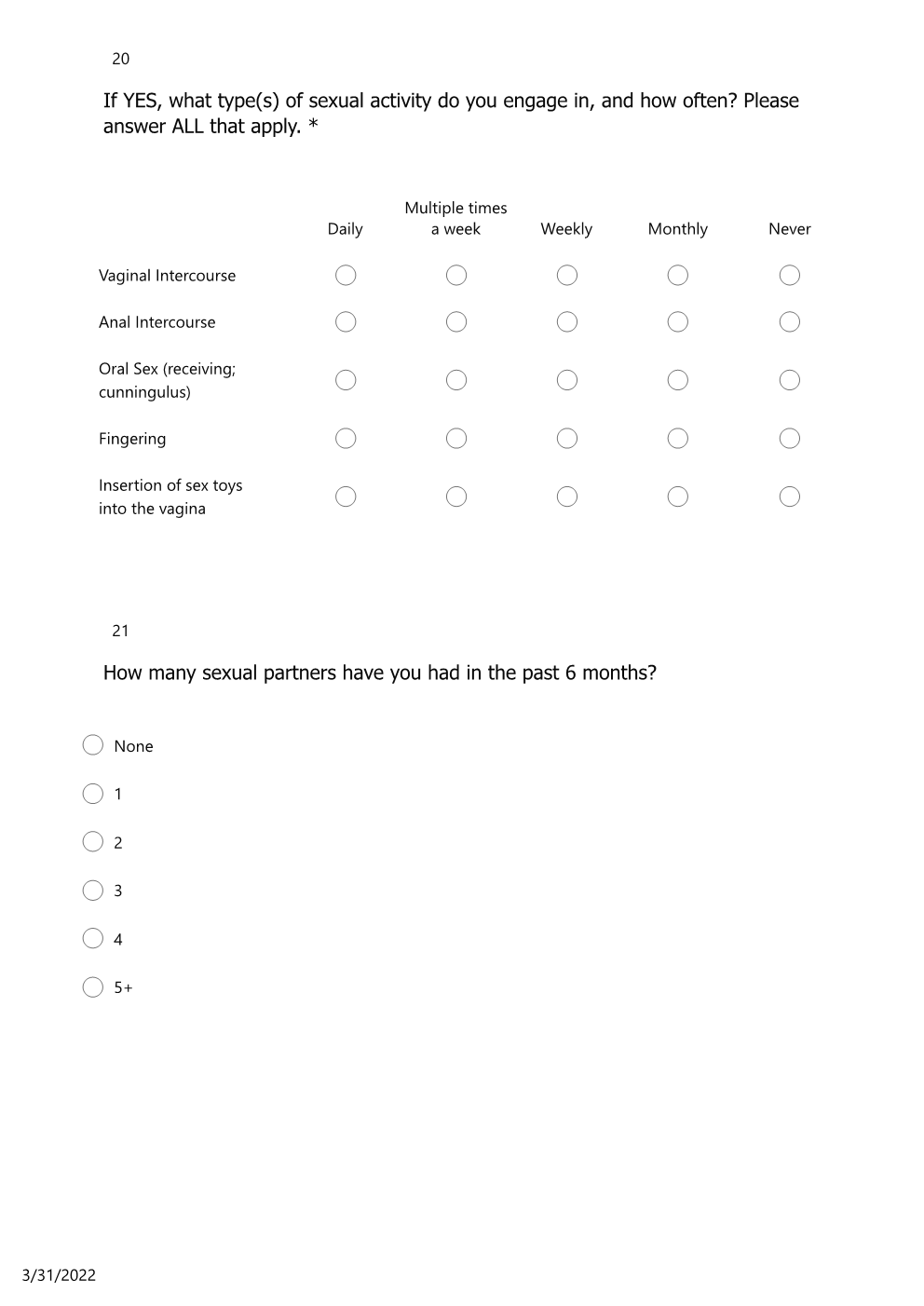

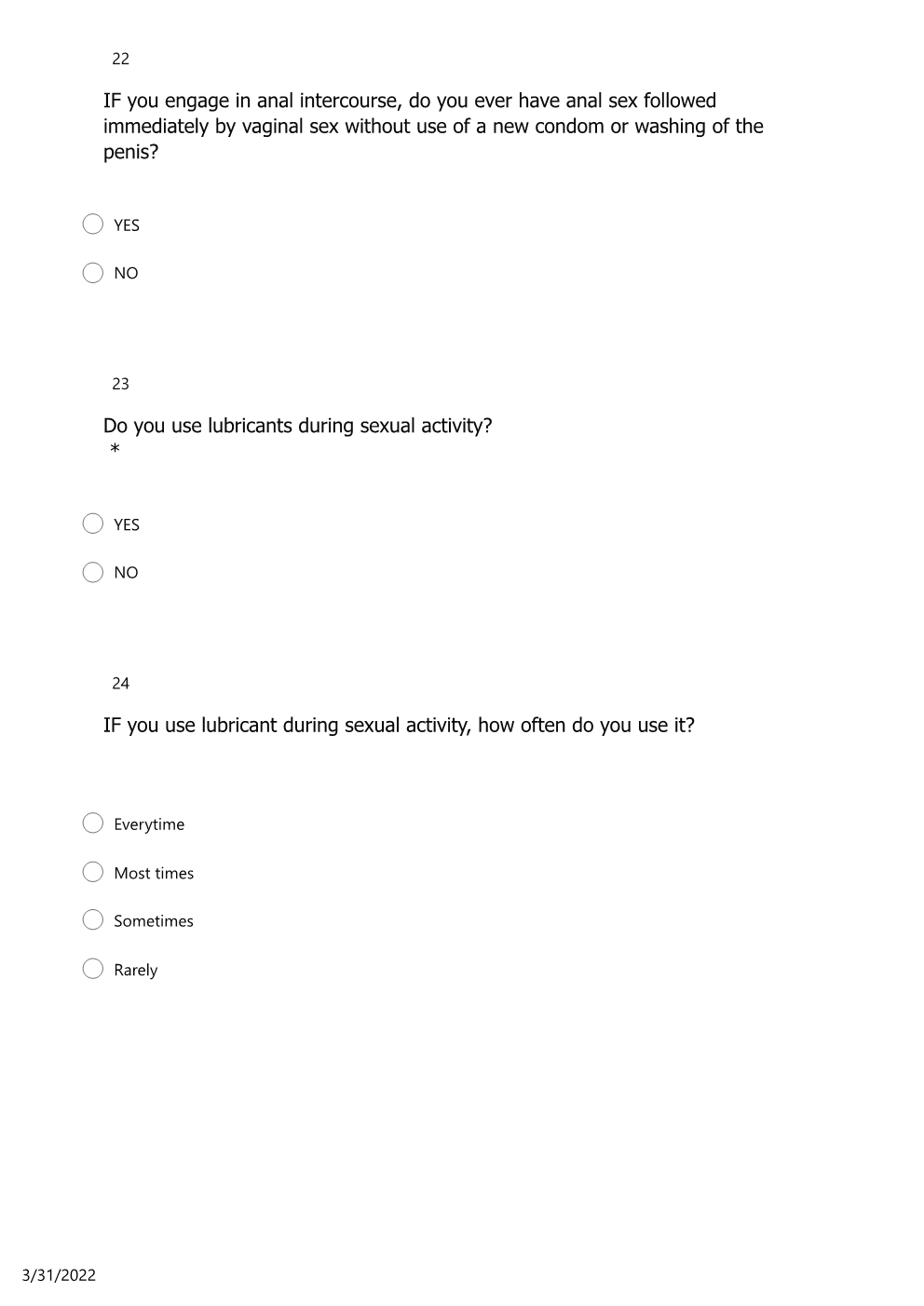

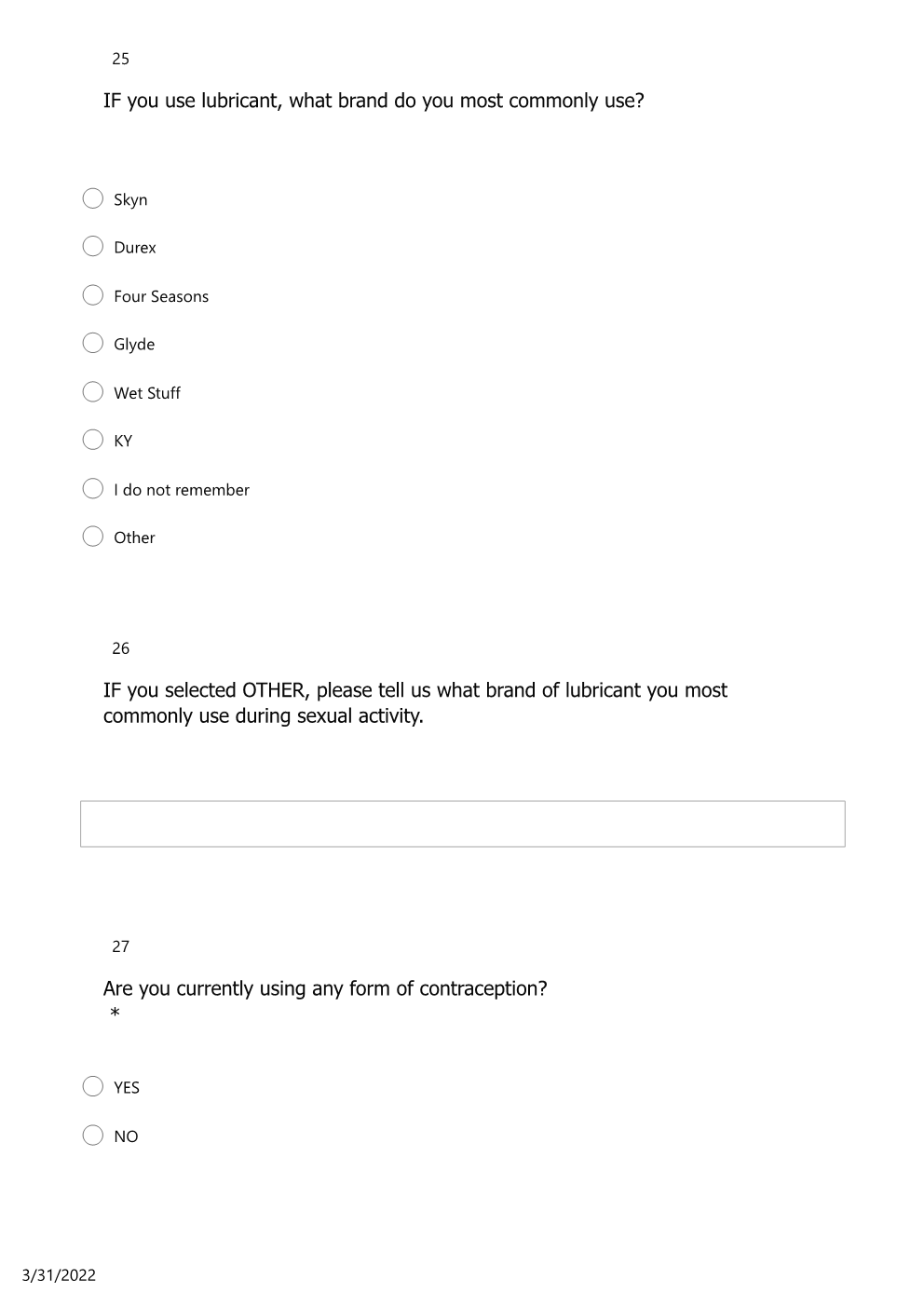

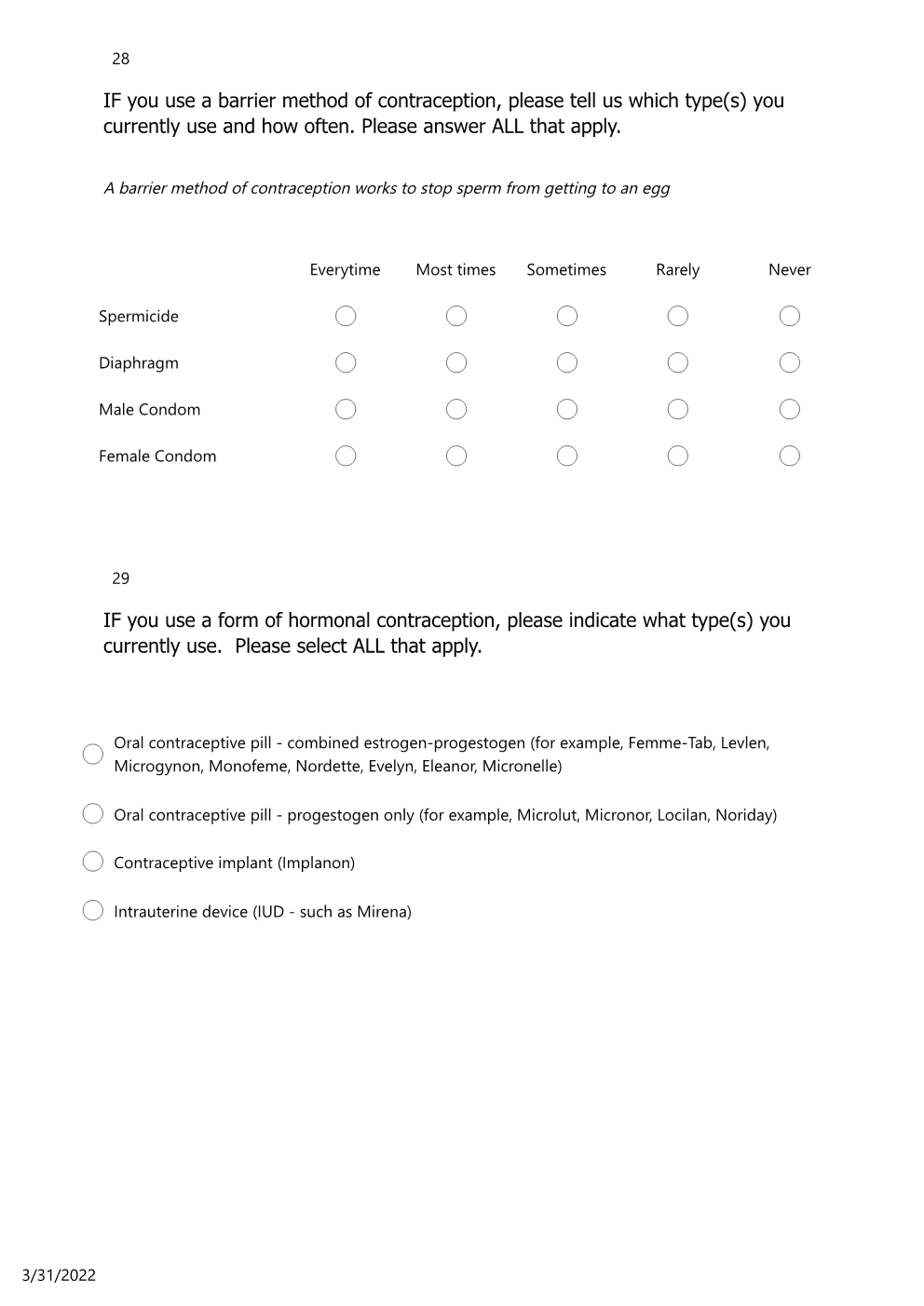

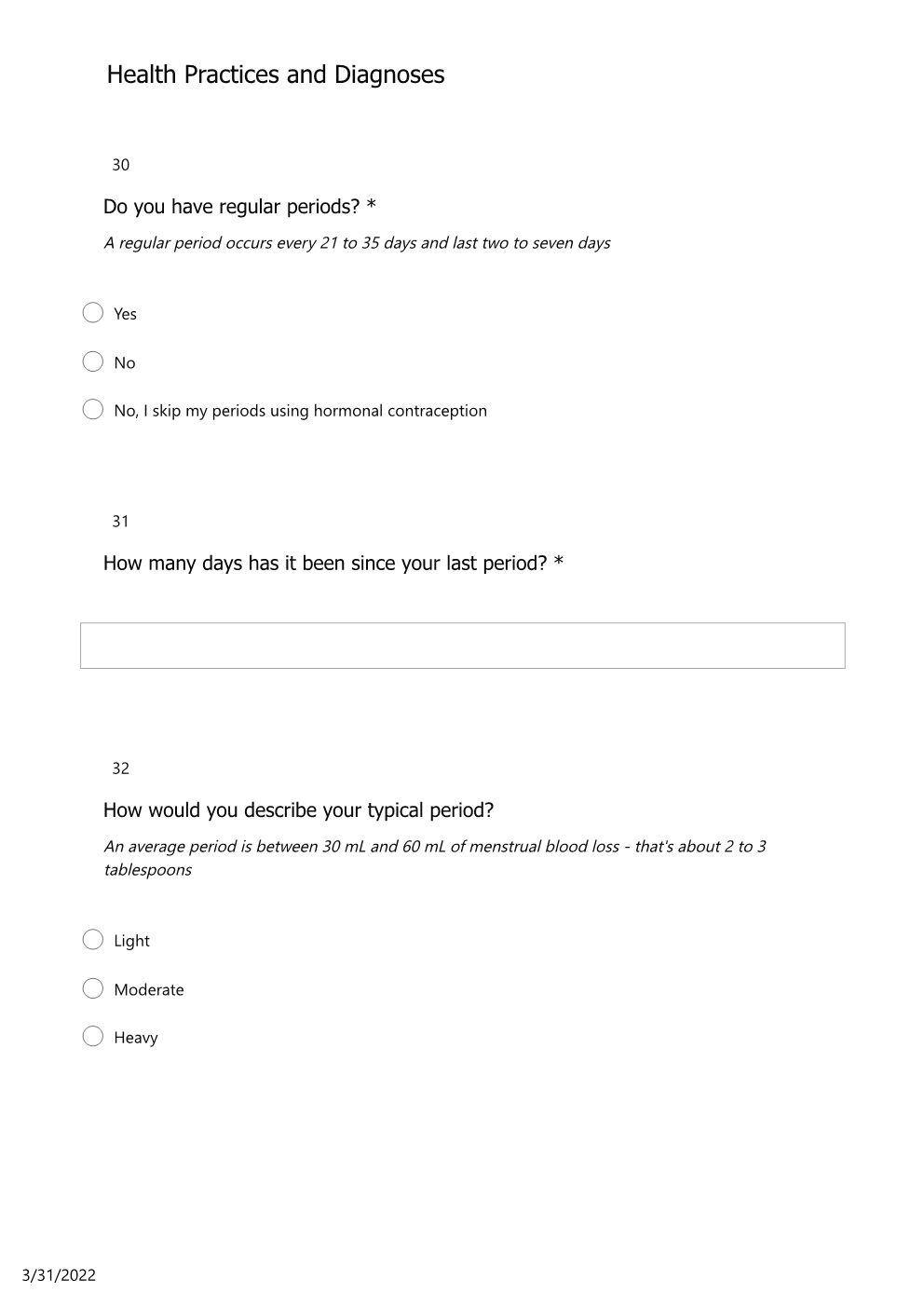

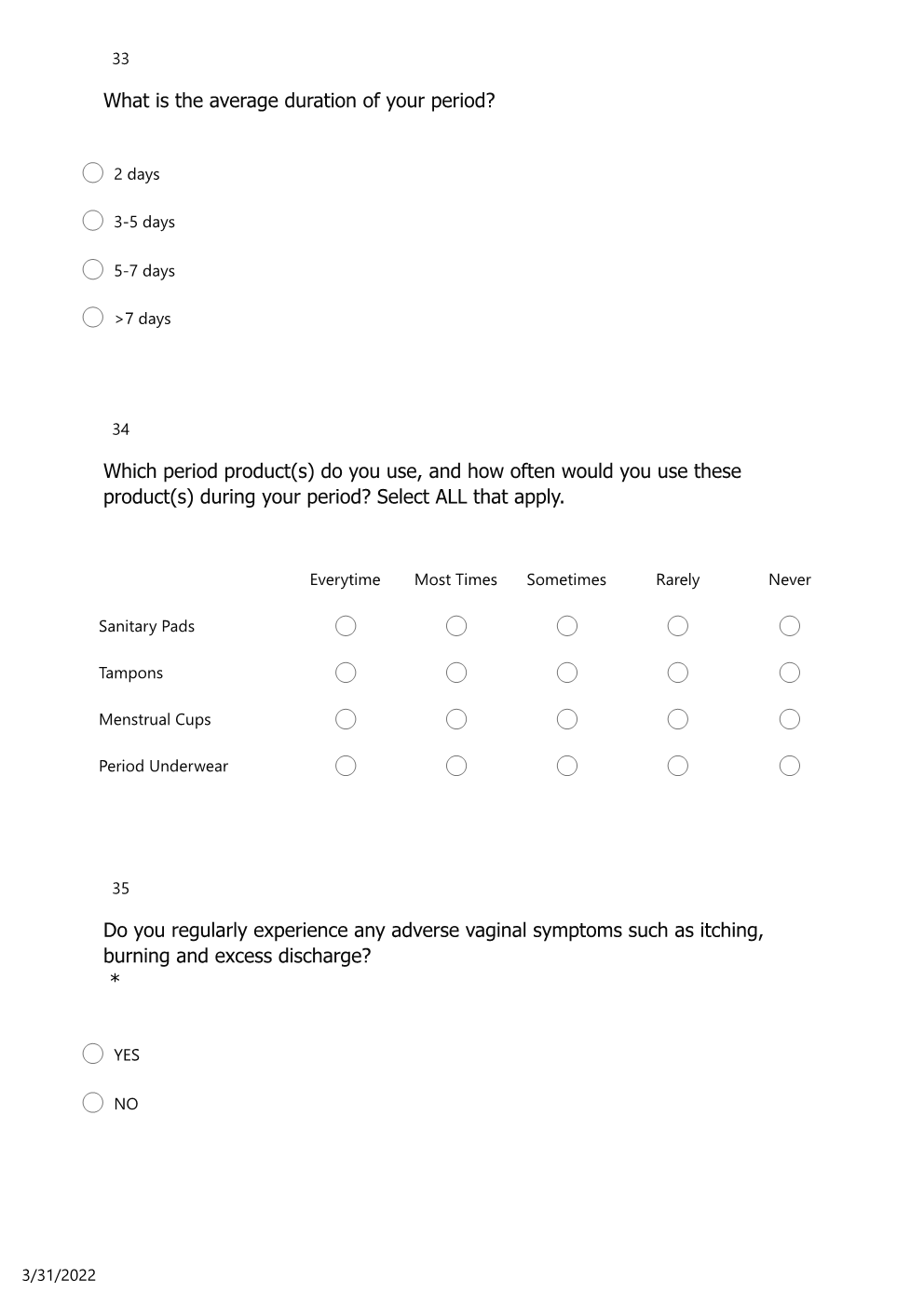

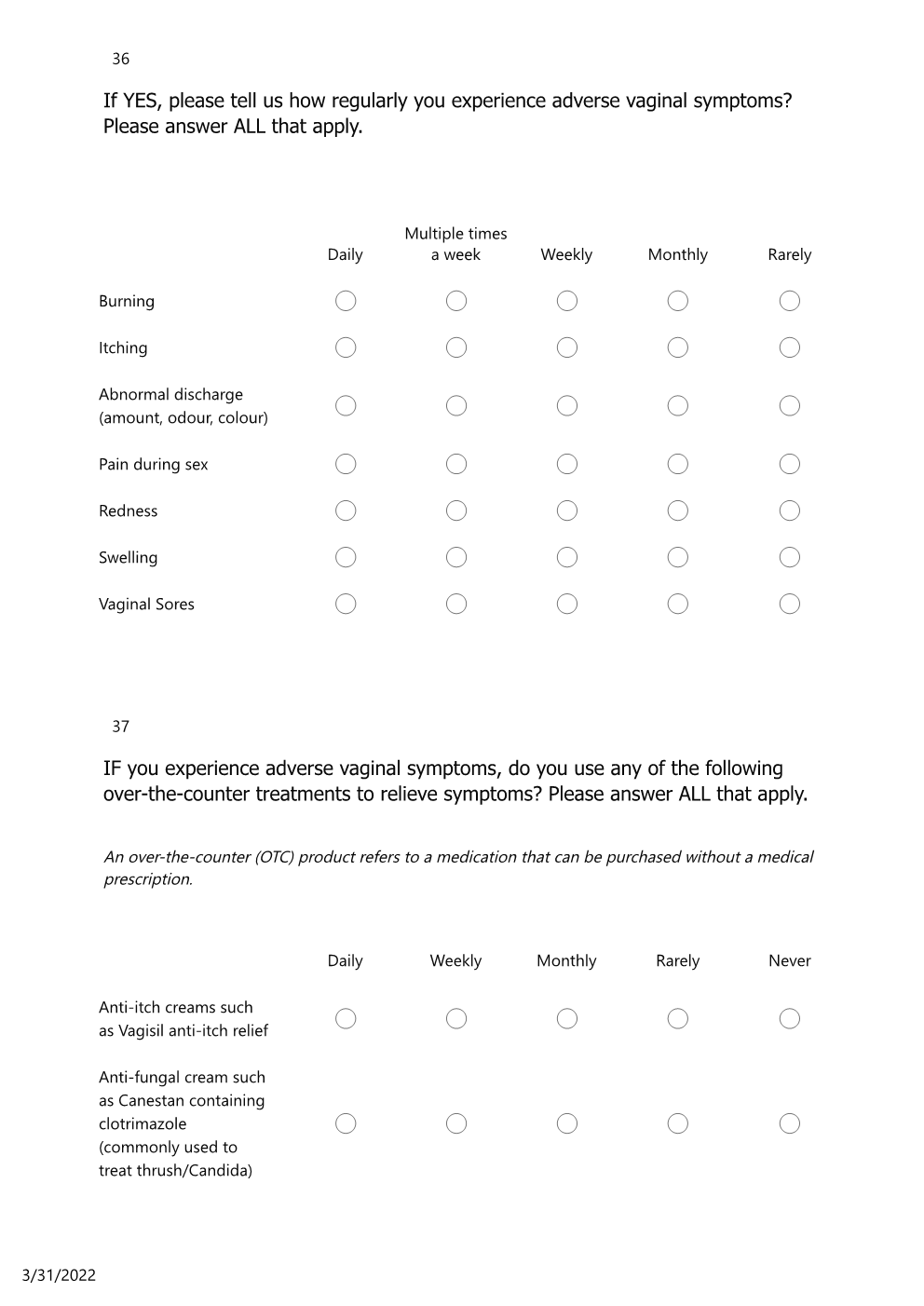

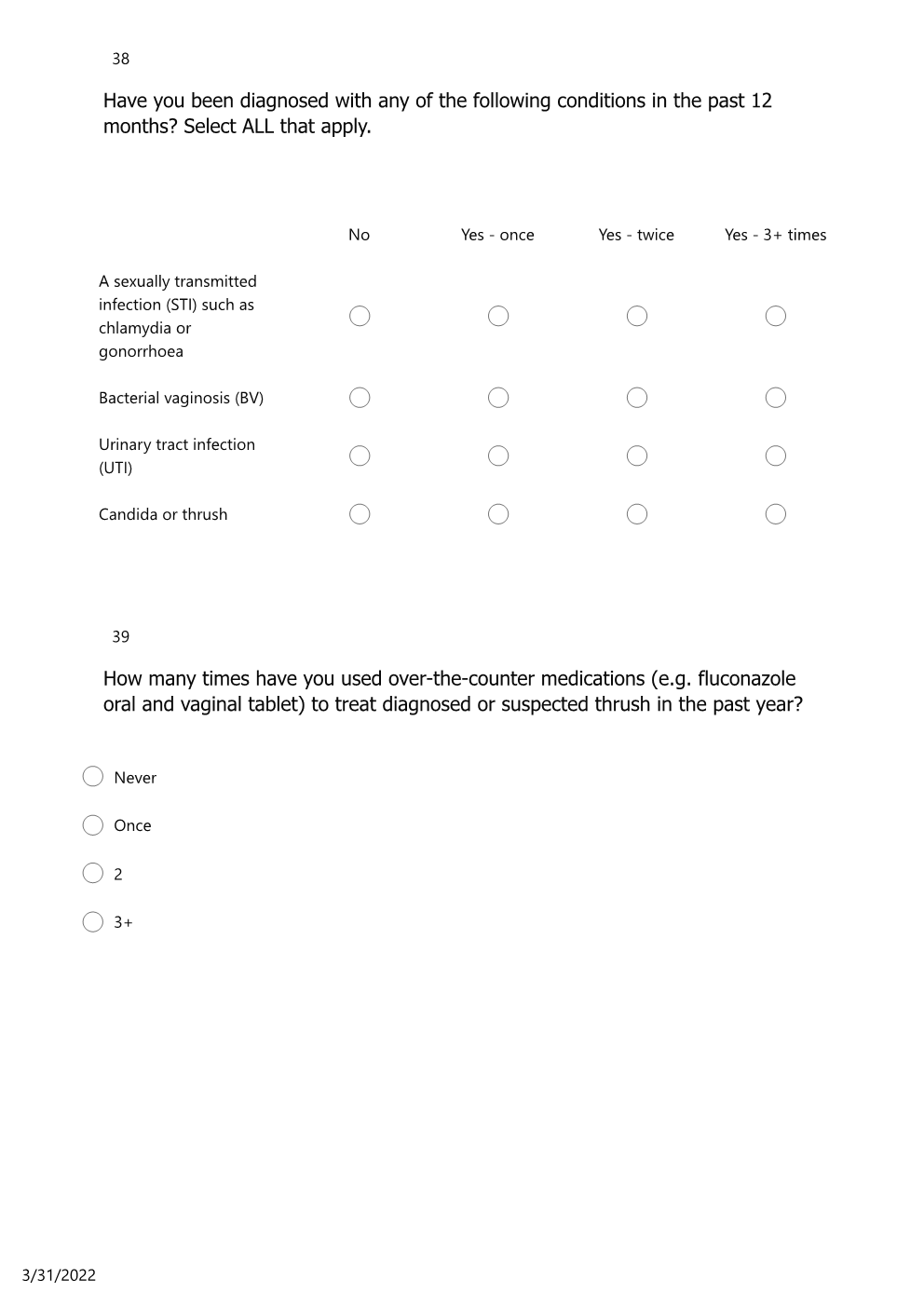

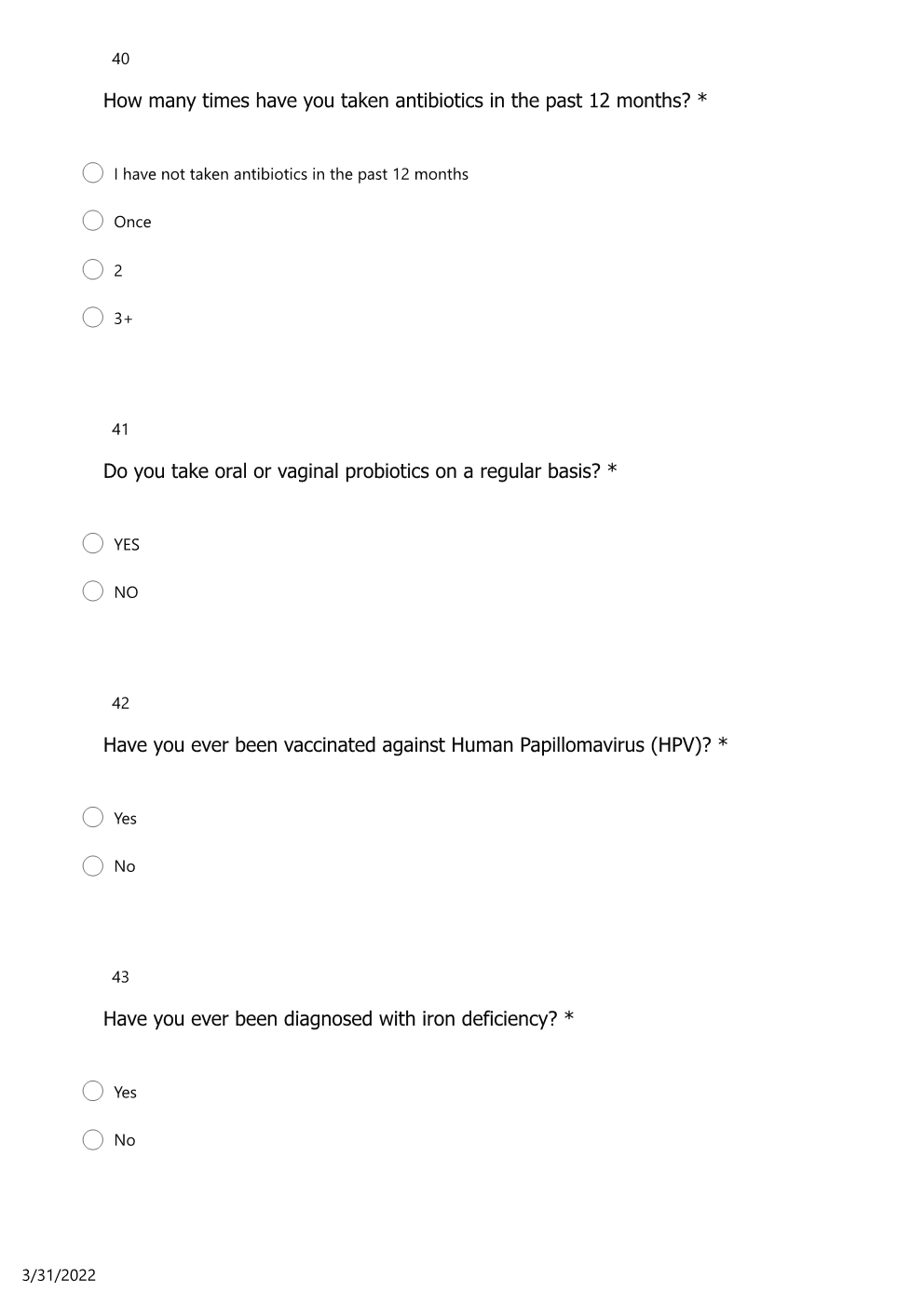

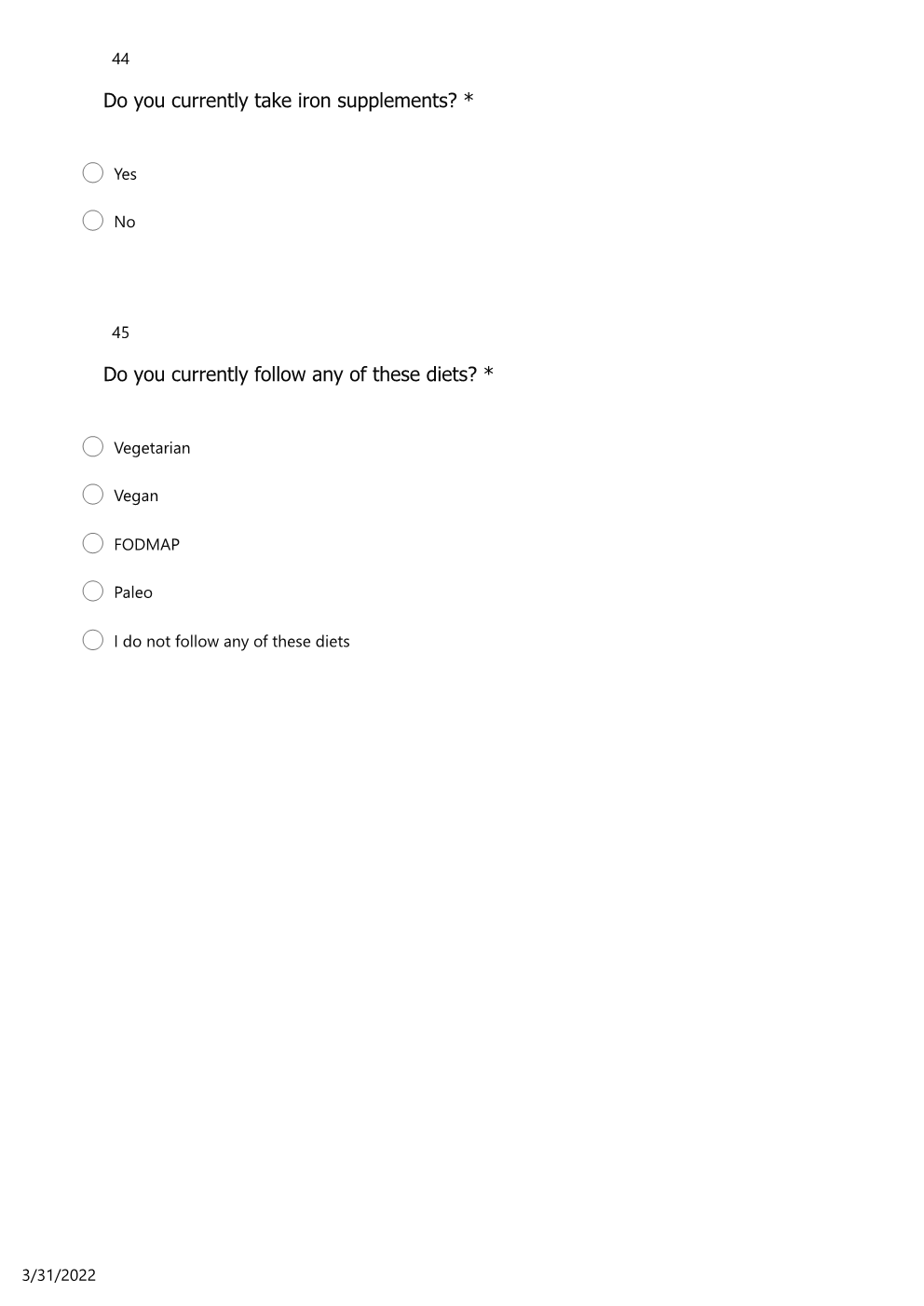
**

**
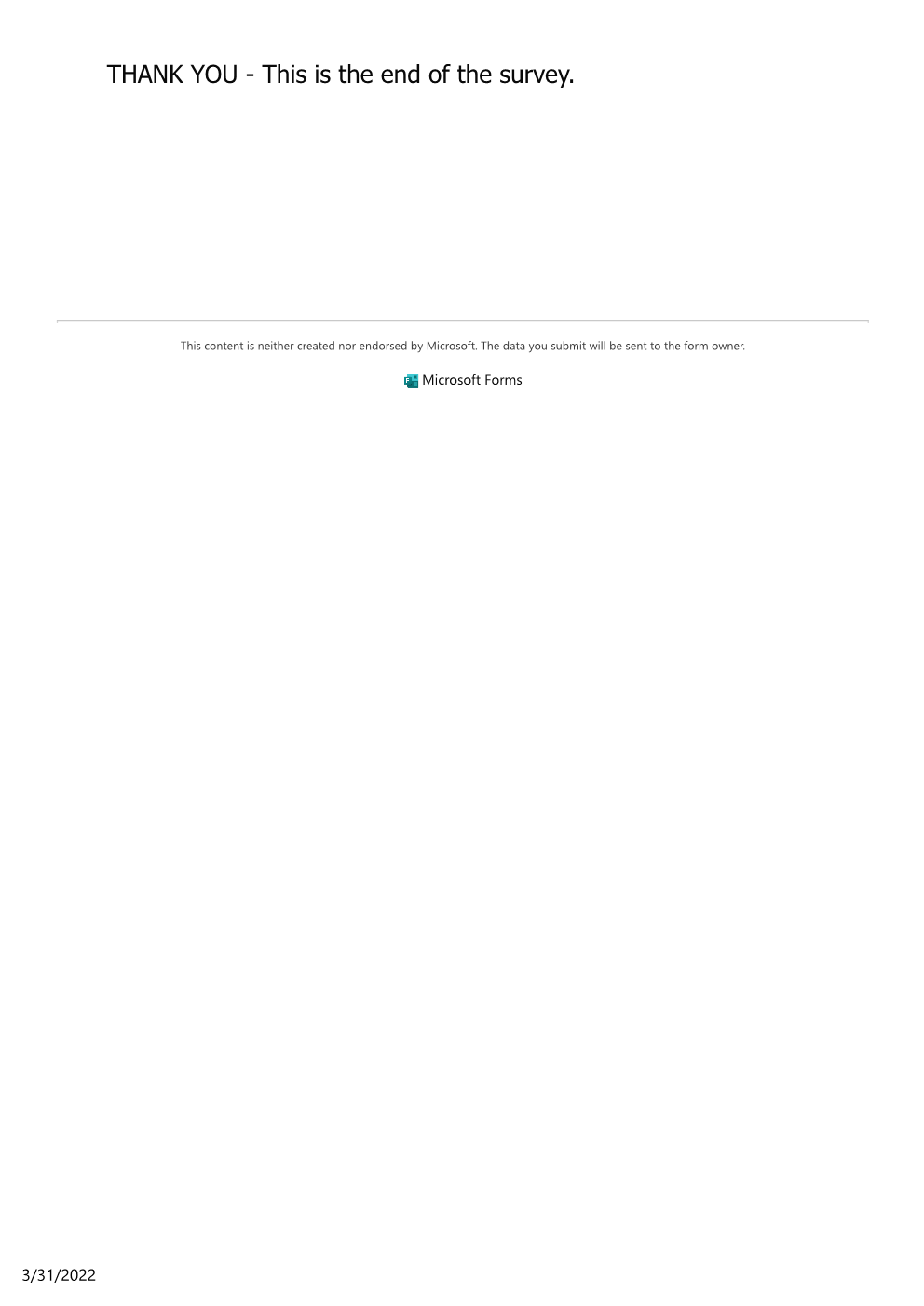
**

Supplement: Supplementary file 2 — Appendix S1: Feminine health and hygiene practices survey. [file RMB2-24-e12685-s002.docx]
